# Decarboxylation and Tandem Reduction/Decarboxylation Pathways to Substituted Phenols from Aromatic Carboxylic Acids Using Bimetallic Nanoparticles on Supported Ionic Liquid Phases as Multifunctional Catalysts

**DOI:** 10.1021/jacs.3c09290

**Published:** 2023-10-10

**Authors:** Natalia Levin, Lisa Goclik, Henrik Walschus, Neha Antil, Alexis Bordet, Walter Leitner

**Affiliations:** † Max Planck Institute for Chemical Energy Conversion, Stiftstr. 34-36, 45470 Mülheim an der Ruhr, Germany; ‡ Institut für Technische und Makromolekulare Chemie, RWTH Aachen University, Worringerweg 2, 52074 Aachen, Germany

## Abstract

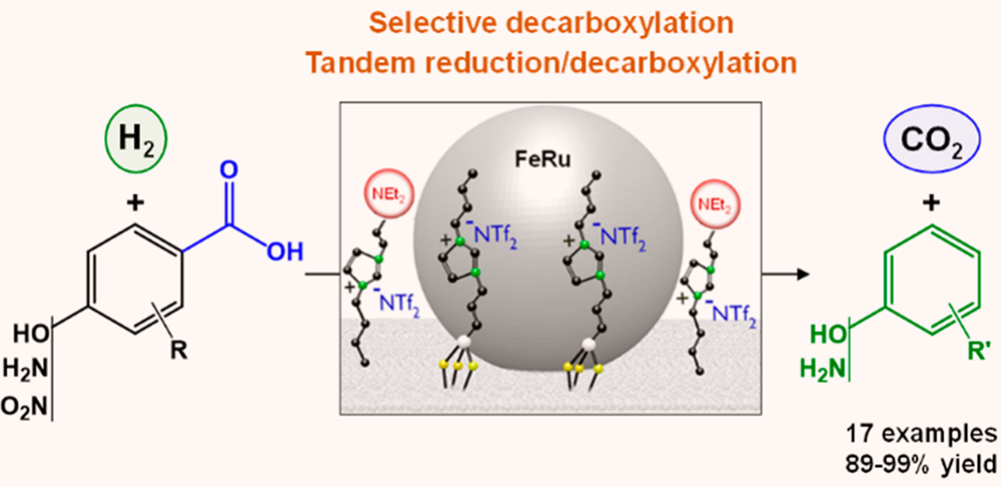

Valuable substituted phenols are accessible via the selective decarboxylation of hydroxybenzoic acid derivatives using multifunctional catalysts composed of bimetallic iron–ruthenium nanoparticles immobilized on an amine-functionalized supported ionic liquid phase (Fe_25_Ru_75_@SILP+IL-NEt_2_). The individual components of the catalytic system are assembled using a molecular approach to bring metal and amine sites into close contact on the support material, providing high stability and high decarboxylation activity. Operating under a hydrogen atmosphere was found to be essential to achieve high selectivity and yields. As the catalyst materials enable also the selective hydrogenation and hydrodeoxygenation of various additional functional groups (i.e., formyl, acyl, and nitro substituents), direct access to the corresponding phenols can be achieved via integrated tandem reactions. The approach opens versatile synthetic pathways for the production of valuable phenols from a wide range of readily available substrates, including compounds derived from lignocellulosic biomass.

## Introduction

Phenol and its derivatives such as alkylphenols and aminophenols are important compounds that find widespread application in all areas of the chemical industry, including the production of commodities, fine chemicals, agrochemicals, and pharmaceuticals.^1−9^ Nowadays, more than 99% of the world’s phenol production (11 million tons in 2018) arises from the cumene process, where the petrochemical feedstocks benzene and propene are converted into phenol and acetone.^3^ Alkylphenols are typically produced through the direct alkylation of phenol derivatives with olefins.^3,10^ However, this pathway suffers from limited substrate scope.^11−14^ Alternatively, the direct alkylation of phenol with alkyl halides requires harsh conditions and offers poor chemo- and regioselectivity.^10^ The selective amination of phenol to produce aminophenols also remains a challenge.^15−18^ Therefore, alternative methods to synthesize phenols have been actively researched. For example, the catalytic cleavage of lignin-derived diaryl ethers and lignin model compounds can lead to alkylphenols but current catalytic systems still suffer from low selectivity,^19−22^ narrow substrate scope,^22−24^ low catalyst stability,^21^ or poor recyclability.^24^ The selective reduction of vinylphenols has also been proposed but is hampered by the limited access and high costs of the starting materials.^25,26^

**Figure 1:**
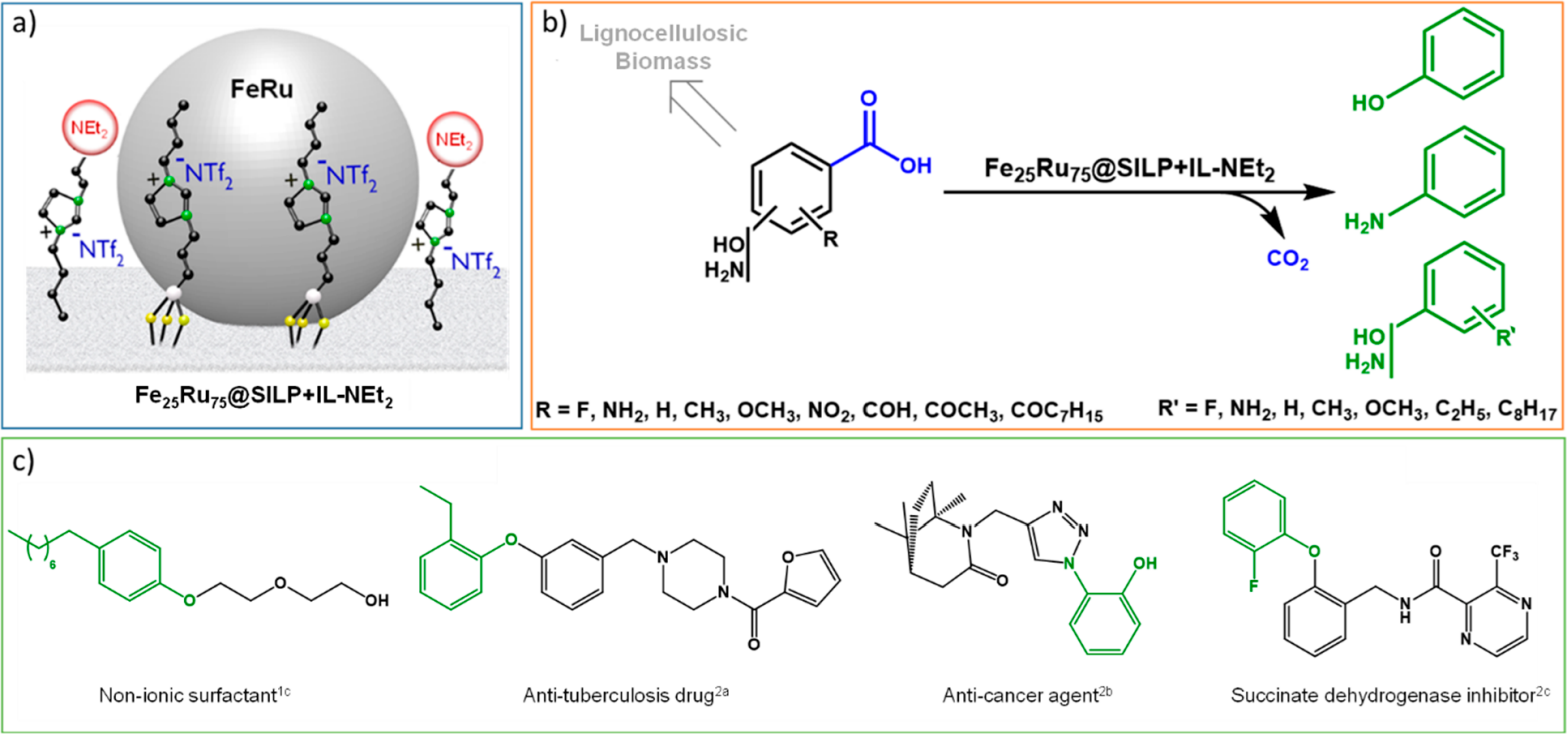
General approach in this study. (a) Illustration of the bimetallic bifunctional Fe_25_Ru_75_@SILP+IL-NEt_2_ catalyst, (b) proposed pathway for the synthesis of phenols through selective decarboxylation of hydroxybenzoic acid derivatives, and (c) examples of applications for phenol derivatives in pharmaceutical products.

In this context, we envisaged to access phenol, alkylphenols, and aminophenols through the selective decarboxylation of hydroxybenzoic acid derivatives (HBAs), including substrates that can be obtained from the oxidative depolymerization of lignin or through biosynthesis^27^ such as 4-hydroxybenzoic acid, vanillic acid, and syringic acid.^28−30^ In addition, the tandem combination of the selective decarboxylation with hydrogenation/hydrodeoxygenation of easily accessible acyl, formyl, and nitro-functionalized HBAs^31−33^ was explored in order to obtain alkylphenols and aminophenols. The required multifunctional catalytic system was designed on the basis of bimetallic FeRu nanoparticles supported on silica modified with amino-substituted ionic liquids (Fe_25_Ru_75_@SILP+IL-NEt_2_) (Figure 1).

## Results and Discussion

### Catalyst Design

Unlocking the potential of the proposed synthetic pathways requires multifunctional catalytic systems capable of decarboxylating and hydrogenating and hydrodeoxygenating substrates while leaving the aromatic ring untouched. Selective decarboxylation and reduction processes have already been investigated individually; however, to the best of our knowledge, there is no existing approach capable of combining them in a single catalytic system. The decarboxylation of aromatic acids is known to be an exergonic reaction favored entropically at low partial pressures of CO_2_. The Δ*G* of the reaction of decarboxylation of 4-hydroxybenzoic acid was estimated to be −17.7 kcal·mol^–1^ through density functional theory (DFT) calculations (see Table S1 in the Supporting Information for details). The model used was validated by comparing values estimated for benzoic acid and chlorobenzoic acid to published data (Table S1).^34,35^ While decarboxylation of aromatic carboxylic acids is typically performed using transition-metal catalysts^36−42^ based on metals such as Cu,^37,38^ Ag,^39^ Au,^40^ Pd,^41^ or Rh,^42^ base-catalyzed decarboxylation reactions using amines were also reported for aromatic substrates bearing appropriate functional groups stabilizing the intermediate carbanion.^43−45^ At present, however, examples of selective decarboxylation of HBAs are scarce in the literature, and limited to narrow substrate scopes and low yields.^20,37,45−48^ For example, attempts to decarboxylate 4-HBA, vanillic acid, and syringic acid using Cu-based catalysts^20^ or ILs^47,48^ gave only yields up to 50%. On the other hand, metal nanoparticles immobilized on molecularly modified surfaces^49^ have been shown to act as excellent catalysts for hydrogenation,^50−57^ hydrodeoxygenation^58−62^ and hydrogenolysis reactions.^19^ The combination of noble metals with excellent hydrogenation properties (e.g., Ru, Rh, etc.) with their more oxophilic 3d congeners (e.g., Fe, Co, etc.) was recently shown to produce bimetallic NPs with enhanced C=O hydrogenation activity, while suppressing aromatic ring hydrogenation.^49,50,53,57^ In particular, the use of bimetallic FeRu-nanoparticles on ionic-liquid-modified silica support (Fe_25_Ru_75_@SILP) catalyst was found capable of hydrodeoxygenating acetophenone derivatives to alkylated aromatic products.^59,60^ The flexibility of the NPs@SILP catalytic platform for further customization prompted us to attempt the combination of FeRu NPs with the amine-functionalized SILP to integrate decarboxylation and hydrogenation capacity in a single material (Figure 1a).

### Catalyst Synthesis and Characterization

The new multifunctional catalyst Fe_25_Ru_75_@SILP+IL-NEt_2_ was assembled in a versatile manner through the physisorption of the basic ionic liquid 1-[2-(diethylamino)ethyl]-3-butylimidazolium bis(trifluoromethane)sulfonamide (IL-NEt_2_) on the previously reported Fe_25_Ru_75_@SILP material.^50^ In brief, the preparation of Fe_25_Ru_75_@SILP involved the condensation of [1-butyl-3-(3-triethoxy-silylpropyl)-imidazolium]NTf_2_ with dehydroxylated SiO_2_. This molecular modifier is a typical imidazolium-based IL used in many precedent studies,^50−53,58−60^ and the NTf_2_ anion was selected for its stability, hydrophobicity, low nucleophilicity, non-coordinating, and redox innocent nature.^63−66^ The synthetic procedure continues with the *in situ* reduction of a mesitylene solution of {Fe[N(Si(CH_3_)_3_)_2_]_2_}_2_ and [Ru(cod)(cot)] (cod = cyclooctadiene; cot = cyclooctatriene) under an atmosphere of H_2_ (3 bar) at 150 °C (for the detailed procedure, see the Supporting Information). The oxidation state and alloy extent of the Fe_25_Ru_75_ NPs in Fe_25_Ru_75_@SILP were previously studied by XANES and EXAFS, evidencing zerovalent Fe and Ru atoms organized in a homophilic bimetallic structure.^50^ The newly synthesized amine functionalized IL-NEt_2_ showed basic properties in water, with an experimentally determined p*K*_aH_ value of 7.21 ± 0.01 (expressed as the strength of its conjugated acid) (Figure S1), consistent with DFT calculations (7.3, Table S2). Its physisorption to prepare the bifunctional catalyst (Fe_25_Ru_75_@SILP+IL-NEt_2_) was achieved by stirring a suspension of Fe_25_Ru_75_@SILP and IL-NEt_2_ in acetone at room temperature, followed by removal of the solvent under a vacuum.^58^ After varying the loading of physisorbed IL-NEt_2_ (Table S3), it was fixed to 0.731 ± 0.001 mmol·g^–1^ for the rest of the study, corresponding to an IL-NEt_2_/metal molar ratio of 2.9.

**Figure 2:**
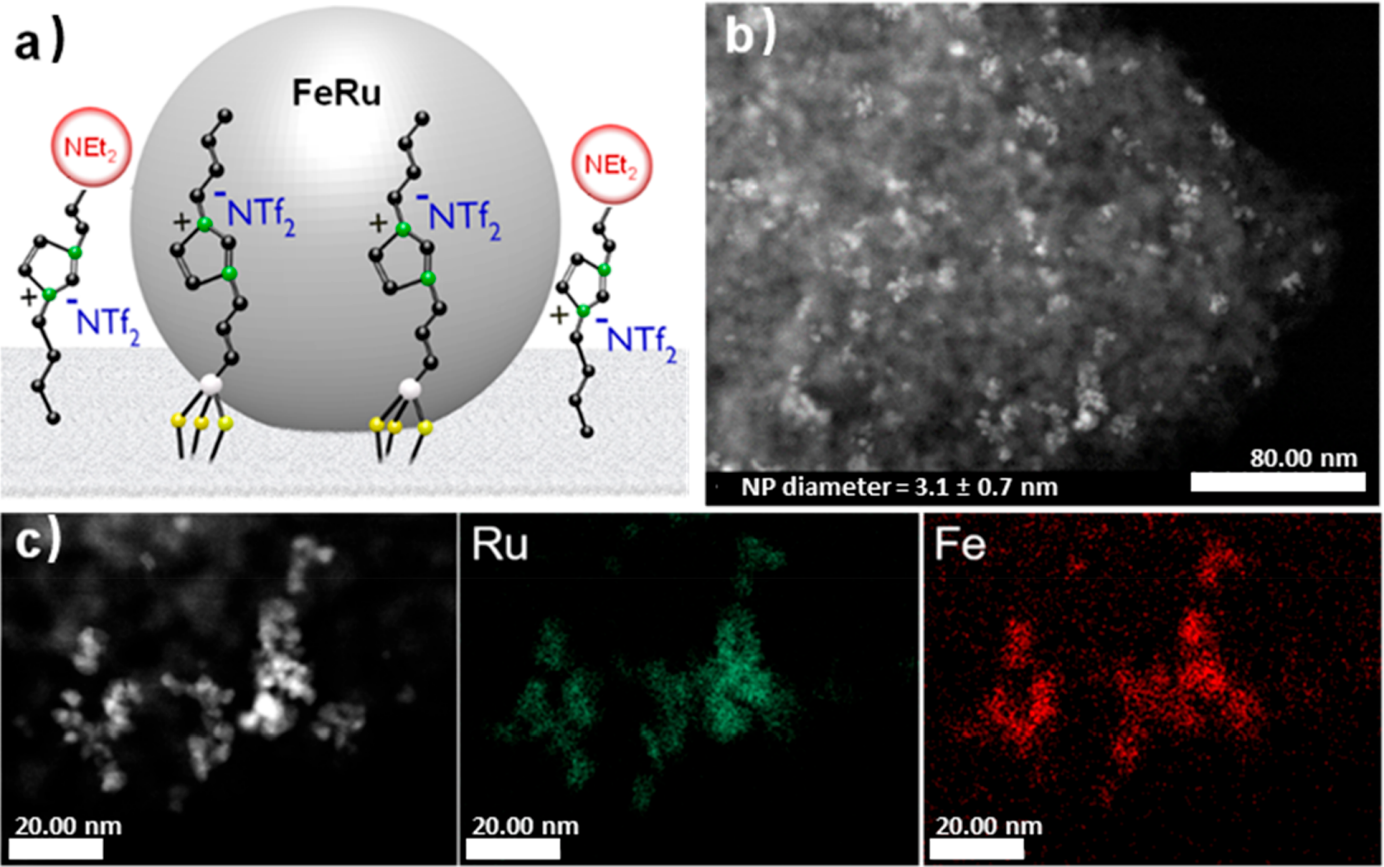
Fe_25_Ru_75_@SILP+IL-NEt_2_ bimetallic bifunctional catalytic system. (a) Illustration, (b) STEM-HAADF image, (c) STEM-HAADF-EDS elemental mapping (green = Ru, red = Fe).

As expected, the BET surface area (36 m^2^·g^–1^) and the pore volume (0.13 m^3^·g^–1^) measured by N_2_ adsorption experiments were significantly reduced upon the physisorption of IL-NEt_2_ on Fe_25_Ru_75_@SILP (Table S4). Titration of the amount of accessible amine functionalities on Fe_25_Ru_75_@SILP+IL-NEt_2_ gave 0.49 ± 0.01 mmol·g^–1^, which corresponds to 66.4 ± 0.1% of the theoretical total amount for quantitative adsorption. Characterization of Fe_25_Ru_75_@SILP+IL-NEt_2_ by scanning transmission electron microscopy in high angle annular dark field (STEM-HAADF) associated with energy dispersive X-ray spectroscopy (EDS) showed the presence of small (3.1 nm) bimetallic NPs containing Fe and Ru (Figure 2).

Scanning electron microscopy (SEM) with EDS confirmed the expected metal ratio and metal loading (0.34 ± 0.03 mmol·g^–1^, Table S4). Thus, physisorption did not affect significantly the metal loading or ratio, nor the size of the metal NPs, even though they were slightly more aggregated than on the pristine Fe_25_Ru_75_@SILP material.^50^

### Catalytic Study

#### Identification of Reaction Parameters

The catalytic performances of the bifunctional Fe_25_Ru_75_@SILP+IL-NEt_2_ catalyst as well as several reference materials were first investigated for the decarboxylation of 4-hydroxybenzoic acid (**1**) as a model reaction (Table 1). The standard conditions were set to 175 °C, 18 h, heptane as solvent, and 65 equiv of substrate with respect to the total metal loading (30 equiv relative to the amine groups of IL-NEt_2_). Performing this reaction without applying any pressure resulted in an extremely low mass balance (Table 1, entry 1). Using 50 bar of inert gas pressure (N_2_ or Ar) allowed observing significant conversion of **1** to phenol (**1a**) yet still with low yields (18% and 10%, respectively) and unclosed mass balances (Table 1, entries 2 and 3). In contrast, **1** was fully and selectively decarboxylated using Fe_25_Ru_75_@SILP+IL-NEt_2_ under a H_2_ atmosphere (50 bar), affording **1a** in quantitative yield without reduction of the aromatic moiety (Table 1, entry 4).

**Table 1:**
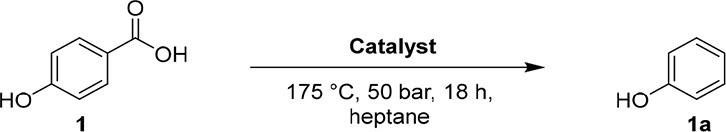
Decarboxylation of **1** Using Fe_25_Ru_75_@SILP+IL-NEt_2_ and Various Reference Materials^a^

**#**	**Catalyst**	**Applied gas**	***X* [%]**	***Y***_**1a**_**[%]**
1	Fe_25_Ru_75_@SILP+IL-NEt_2_	None	-	-
2	Fe_25_Ru_75_@SILP+IL-NEt_2_	N_2_	18 ± 5	18 ± 5
3	Fe_25_Ru_75_@SILP+IL-NEt_2_	Ar	10	10
4	Fe_25_Ru_75_@SILP+IL-NEt_2_	H_2_	>99	>99
5	None	H_2_	0	0
6	Fe_25_Ru_75_@SILP	H_2_	4 ± 1	4 ± 1
7	SILP+IL-NEt_2_	H_2_	58 ± 2	58 ± 2
8	Fe_40_Ru_60_@SILP+IL-NEt_2_	H_2_	33 ± 2	33 ± 2
9	Fe_60_Ru_40_@SILP+IL-NEt_2_	H_2_	37 ± 1	37 ± 1
10	Fe_100_@SILP	H_2_	0	0
11	Ru_100_@SILP	H_2_	>99	0^b^
12	Fe_25_Ru_75_@SiO_2_	H_2_	>99	39^c^
13	Fe_25_Ru_75_@SILP+IL-NEt_2_^d^	H_2_	>99	>99
14	Fe_25_Ru_75_@SILP+IL-SO_3_H	H_2_	0	0
15	Fe_25_Ru_75_@SILP+IL-NH_2_	H_2_	6	6
16	Fe_25_Ru_75_@SILP + SILP+IL-NEt_2_	H_2_	68 ± 3	68 ± 3
17	Fe_25_Ru_75_@SILP+IL-NEt_2_	D_2_^e^	>99	>99

a Standard reaction conditions: Catalyst (metal content: 0.0034 mmol), substrate (0.221 mmol, 65 equiv compared to metal), solvent = heptane (0.5 mL), 175 °C, H_2_ (50 bar), 18 h, 500 rpm; *X* = conversion, *Y* = yield, determined by GC-FID using tetradecane as internal standard. Variations are shown for two independent experiments.

b Product = cyclohexanol (99%).

c Byproducts = cyclohexanol (38%) and cyclohexane (23%).

d With Br anion.

e 20 bar of D_2_, 200 °C.

Without a catalyst, no conversion was observed under these conditions (Table 1, entry 5). Similarly, using the bimetallic Fe_25_Ru_75_@SILP catalyst without a physisorbed IL resulted in very low decarboxylation activity (4% yield, Table 1, entry 6). With SILP+IL-NEt_2_ in the absence of Fe_25_Ru_75_ NPs, the conversion and yield of **1a** reached only 58% (Table 1, entry 7). Lowering the amount of Ru in the bimetallic particles in Fe_40_Ru_60_@SILP+IL-NEt_2_ and Fe_60_Ru_40_@SILP+IL-NEt_2_ resulted in lower conversions and yields (Table 1, entries 8 and 9). As expected, Fe_100_@SILP gave no activity at all (Table 1, entry 10), while Ru_100_@SILP led to complete hydrogenation of the aromatic ring (Table 1, entry 11). Bimetallic FeRu NPs prepared directly on silica (Fe_25_Ru_75_@SiO_2_) gave very low selectivity toward the formation of **1a** (Table 1, entry 12) accompanied by significant aromatic ring hydrogenation, indicating the presence of monometallic Ru-particles. This corroborates with the previously reported beneficial role of IL-modifiers to avoid segregated particles upon synthesis of bimetallic NPs.^53,57^ Replacing the NTf_2_ anion by Br did not change the product distribution under these conditions (Table 1, entry 13). The recently reported acid-functionalized Fe_25_Ru_75_@SILP+IL-SO_3_H catalyst^58^ led to no reaction, evidencing the need for a base (Table 1, entry 14). Replacing the tertiary amine functionality of IL-NEt_2_ by a primary amine (IL-NH_2_, calculated p*K*_aH_ in water = 4.8, Table S2) led to a loss of catalytic activity (Table 1, entry 15). A physical mixture of Fe_25_Ru_75_@SILP and triethylamine (p*K*_aH_ in water = 10.7) afforded full conversion, while losing the potential for reusability (Table S5). Interestingly, using secondary and primary amines of similarly high basicity (i.e., diethylamine and butylamine p*K*_aH_ in water = 11.0 and 10.6, respectively) was also successful (Table S5). In contrast, lower activity was observed in the presence of a weaker base such as *N*,*N*-dimethylaniline (61% conversion, p*K*_aH_ in water = 5.1), and diphenylamine (p*K*_aH_ in water = 0.8) was found to be inactive (Table S5). These results indicate that the decarboxylation reaction can proceed smoothly in the presence of primary, secondary, or tertiary amine functionalities, as long as their basicity is sufficient.

Notably, a physical mixture of Fe_25_Ru_75_@SILP and metal-free SILP+IL-NEt_2_ also did not reach the level of performance of Fe_25_Ru_75_@SILP+IL-NEt_2_ (68% conversion and yield, Table 1, entry 16), demonstrating the synergistic action induced by the intimate contact of the two functionalities.

In order to get additional insight into the beneficial role of the hydrogen atmosphere in the decarboxylation reaction, the reaction was performed under standard conditions in the presence of D_2_ (20 bar).

Full conversion to phenol was observed in this case as well (Table 1, entry 17), and ^1^H and ^13^C NMR of the isolated product revealed deuterium incorporation in the *ortho* and *para* positions of the hydroxyl functionality (Table S6 and Figure S2), with 78% of the product being deuterated in *para*. Exposing product **1a** to D_2_ under standard reaction conditions led to H/D exchange in the *ortho* position to the hydroxyl functionality only (Table S6 and Figure S2). This demonstrates that the deuterium incorporation in the *para* position occurs as a result of the decarboxylation process at this position. While no significant H/D exchange of the proton at the carboxylic acid functionality was observed within the first 1 h when 22% conversion was achieved, incorporation of D in the product in the *para* position was already very significant (40% of the product, Figure S3). Analysis of the gas phase confirmed the release of CO_2_ (Figure S4). These results indicate that the incorporation of D in the *para* position occurs from the gas phase during the decarboxylation reaction, potentially as a consequence of heterolytic activation of D_2_ by the NPs generating D^+^ assisted by the base.

**Figure 3:**
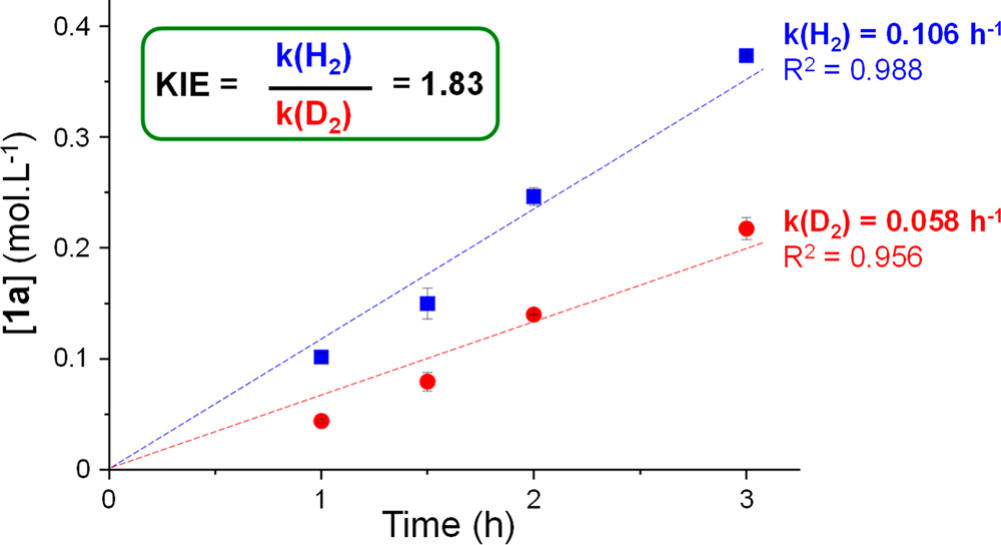
Determination of the kinetic isotope effect for the decarboxylation of **1** using Fe_25_Ru_75_@SILP+IL-NEt_2_. Reaction conditions: Catalyst (10 mg, metal content: 0.0034 mmol), **1** (0.221 mmol, 65 equiv compared to metal), heptane (0.5 mL), 175 °C, H_2_ or D_2_ (20 bar), 500 rpm; yield determined by GC-FID using tetradecane as internal standard. The selectivity is >99% in all cases, so conversion = yield. Data points represent average values, and error bars correspond to standard deviations.

To gain additional insight into the mechanism, time profiles of the decarboxylation of **1** were recorded using Fe_25_Ru_75_@SILP+IL-NEt_2_ under H_2_ and D_2_ (Figure 3). The initial rate of the reaction was found to be noticeably higher under H_2_ than under D_2_, resulting in an apparent normal kinetic isotope effect (KIE) of 1.83. This value is sensibly larger than the theoretical maximum for a secondary KIE (1.4),^67^ pointing toward a primary KIE, and thus toward a rate-determining step dependent on the formation or the cleavage of a bond involving a hydrogen atom coming from the gas phase. Since the activation of gaseous hydrogen is mediated by Fe_25_Ru_75_ NPs, this result is another strong indication of their crucial involvement in the decarboxylation process and supports the existence of a strong synergistic interaction between Fe_25_Ru_75_ NPs and amine functionalities brought in intimate contact.

Interestingly, Lercher and co-workers recently showed that the decarboxylation activity of Pd/C could be enhanced by H_2_ pretreatment.^68^ Their method was focused on aryl-substituted aliphatic carboxylic acids having an α-C–H group, and use of H_2_ during the reaction resulted in undesired aromatic ring hydrogenation.

#### Selective Decarboxylation of Aromatic Carboxylic Acids

Based on these promising results, the Fe_25_Ru_75_@SILP+IL-NEt_2_ catalyst was applied to a scope of benzoic acids possessing hydroxyl, methoxy, and amine substituents (Table 2).

**Table 2:**
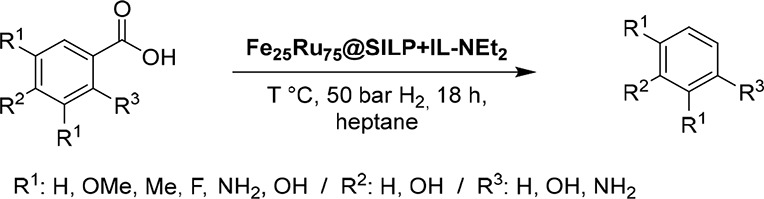

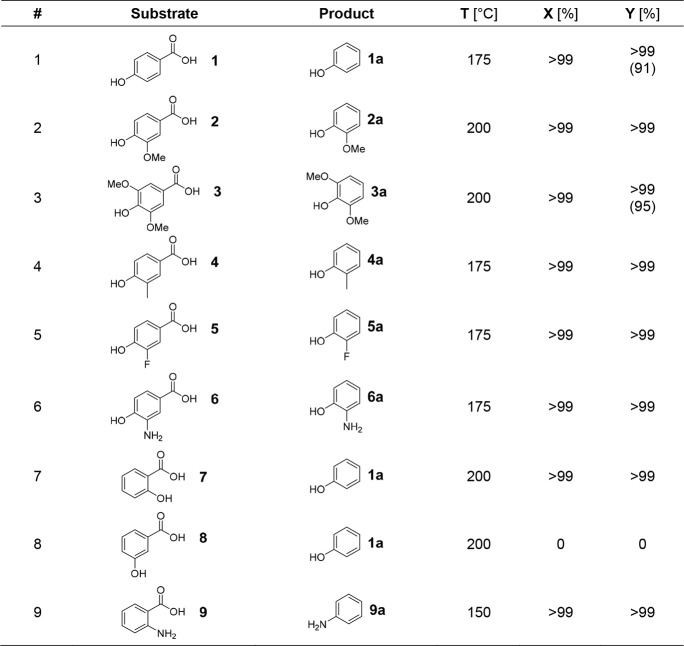
Decarboxylation of Aromatic Carboxylic Acids^a^

a Reaction conditions: Catalyst (10 mg, metal content: 0.0034 mmol), substrate (0.221 mmol, 65 equiv related to total metal loading), solvent = heptane (0.5 mL), H_2_ (50 bar), 18 h, 500 rpm; *Y* = yield, *X* = conversion, determined by GD-FID using tetradecane as internal standard. Isolated yields in parentheses.

Lignin-derived 4-hydroxybenzoic acid derivatives **1**–**3** were readily decarboxylated, giving the corresponding phenols (**1a**–**3a**) in excellent selectivity and yields (Table 2, entries 1–3). Substrates **4**–**6** with electron donating or withdrawing groups in position 3 were also quantitatively converted to the corresponding phenols (Table 2, entries 4–6). Similar results were obtained with salicylic acid (**7**), in which the hydroxyl functionality is in the *ortho* position. In sharp contrast, no conversion was observed when using 3-hydroxybenzoic acid (**8**) as the substrate, suggesting that mesomeric effects are important to observe decarboxylation activity with Fe_25_Ru_75_@SILP+IL-NEt_2_. In the quest for sustainable synthetic pathways for the production of not only phenols but also aniline, the decarboxylation of anthranilic acid is an equally attractive and challenging pathway.^69^ Notably, our catalytic system also showed excellent activity and selectivity for the decarboxylation of anthranilic acid (**9**) to give aniline in quantitative yield (Table 2, entry 9), showing that the decarboxylation activity of Fe_25_Ru_75_@SILP+IL-NEt_2_ is not limited to hydroxybenzoic acid derivatives. In this case, the temperature was reduced to 150 °C to avoid side reactions (e.g., deamination). Straightforward isolation of analytically pure material from the reaction mixtures was demonstrated for products **1a** and **3a** (91 and 95% isolated yield, respectively). Notably, 2,6-dimethoxyphenol (**3a**) is used for the synthesis of cellular antiproliferate active agents and becomes thus accessible from lignin-derived syringic acid.^70^

#### Tandem Reduction and Decarboxylation of Substituted Aromatic Carboxylic Acids

**Figure 4:**
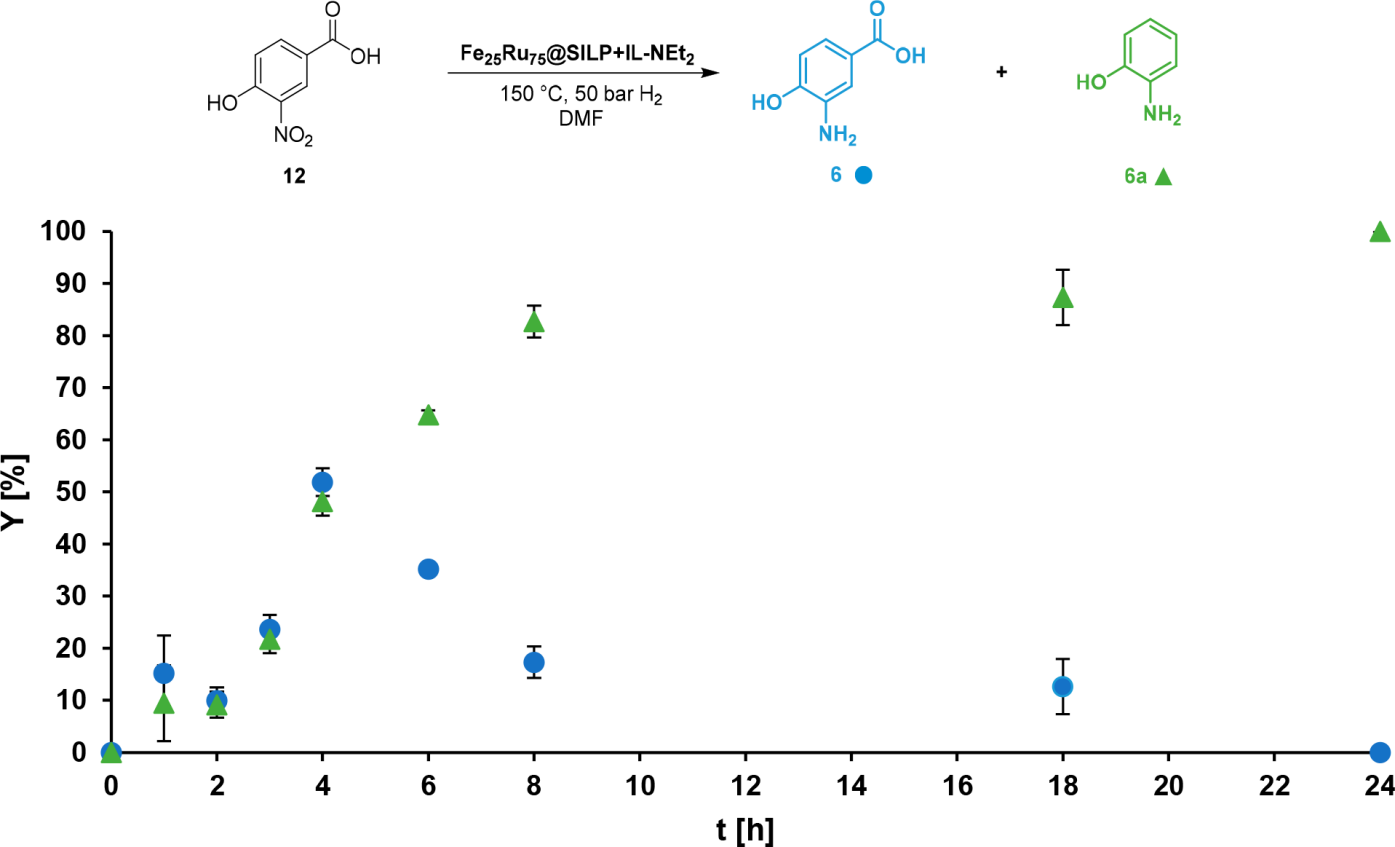
Time profile of the reduction and decarboxylation of 3-nitro-4-hydroxybenzoic acid (**12**) using Fe_25_Ru_75_@SILP+IL-NEt_2_. Conditions: Catalyst (10 mg), substrate = 24.2 mg (30 equiv related to amine, 65 equiv related to total metal loading), 0.5 mL of DMF, 50 bar of H_2_, 150 °C, 500 rpm. *Y* = Yield, determined by GD-FID using tetradecane as internal standard. Error bars represent the standard deviation for two experiments.

Next, the potential of the Fe_25_Ru_75_@SILP+IL-NEt_2_ catalyst for tandem reduction/decarboxylation of hydroxy and aminobenzoic acid derivatives possessing additional reducible functional groups was envisaged to exploit the capacity of hydrogen activation and transfer at the FeRu NPs.^50,59,60^ Under standard conditions, 3-acetyl-4-hydroxybenzoic (**10**) was selectively decarboxylated and hydrodeoxygenated directly to produce 2-ethylphenol (**10a**) in >99% yield (Table 3, entry 1). Following the reduction/decarboxylation for the nitro-substituted HBA **12** as a function of time clearly showed the sequential tandem reaction via 3-amino-4-hydroxybenzoic acid (**6**) as an intermediate (Figure 4). The reaction occurs smoothly to produce selectively 2-aminophenol (**6a**) in nearly quantitative yield (>99%) after 24 h. Adaptation of the standard reaction conditions (temperature, substrate equivalent, solvent) to ensure good solubility and to limit side-reactions allowed efficient conversion of a range of 2- and 4-hydroxybenzoic acid derivatives possessing acetyl, octanoyl, formyl, or nitro substituents (substrates **11**–**16**, Table 3, entries 2–7). The corresponding phenol derivatives (**11a**–**16a**) were produced in excellent selectivity and yields (95–99%) and isolated for selected examples.

**Table 3:**
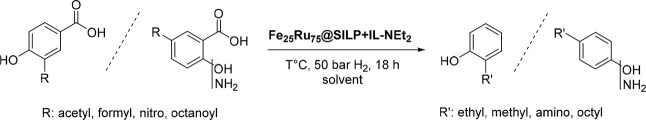

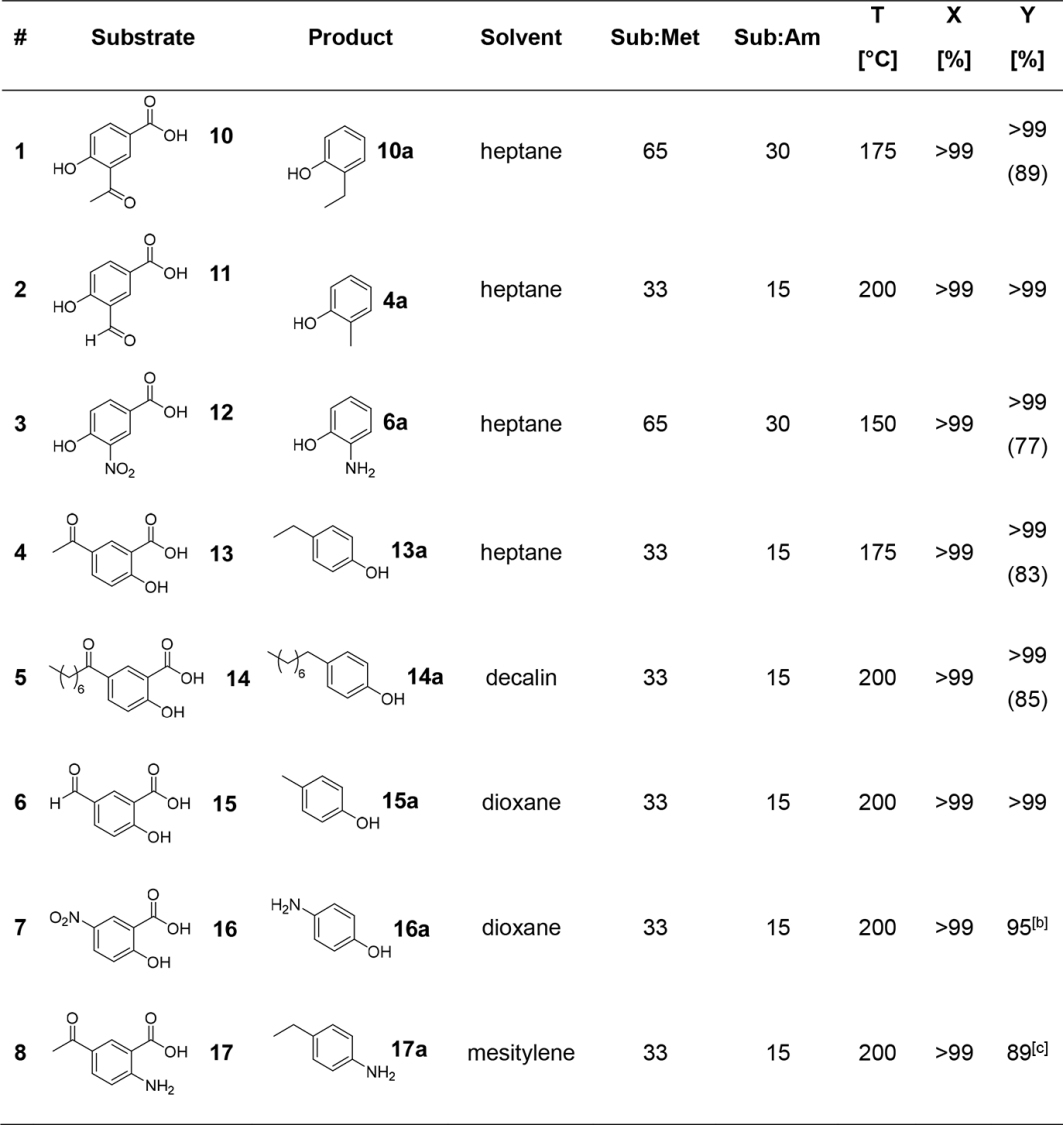
Decarboxylation and Selective Reduction of Aromatic Carboxylic Acids^a^

a Reaction conditions: Catalyst (10 mg, metal content: 0.0034 mmol), solvent = heptane (0.5 mL), H_2_ (50 bar), 18 h, 500 rpm.

b 11% aniline.

c Byproducts: dimers; *Y* = yield, *X* = conversion, determined by GC-FID using tetradecane as internal standard. Isolated yields in parentheses. Sub:Met = substrate to metal ratio, Sub:Am = substrate to amine ratio.

Also, in this case, replacing the hydroxyl by an amine functionality in 2-amino-5-acetylbenzoic acid (**17**) did not influence negatively the catalyst performance, and 4-ethylaniline (**17a**) was obtained in 89% yield (Table 3, entry 8). Interestingly, many of the products formed are valuable chemicals with widespread applications. In particular, **10a**, **6a**, and **17a** are building blocks used in the pharmaceutical industry for the synthesis of anti-tuberculose drugs,^7^ anticancer agents,^8^ and fungicides,^8^ respectively (Figure 1c). **14a** is widely used in the production of nonionic surfactants (Figure 1c).^3^ Using metal-free SILP+IL-NEt_2_ as a catalyst for the conversion of 2-hydroxy-5-octanoylbenzoic acid (**14**) led only to decarboxylation, with 4′-hydroxyoctanophenone as a product (Table S7). This demonstrates the necessity to have Fe_25_Ru_75_ NPs to perform tandem decarboxylation and hydrogenation/hydrodeoxygenation reactions.

#### Catalyst Recycling and Stability

**Figure 5:**
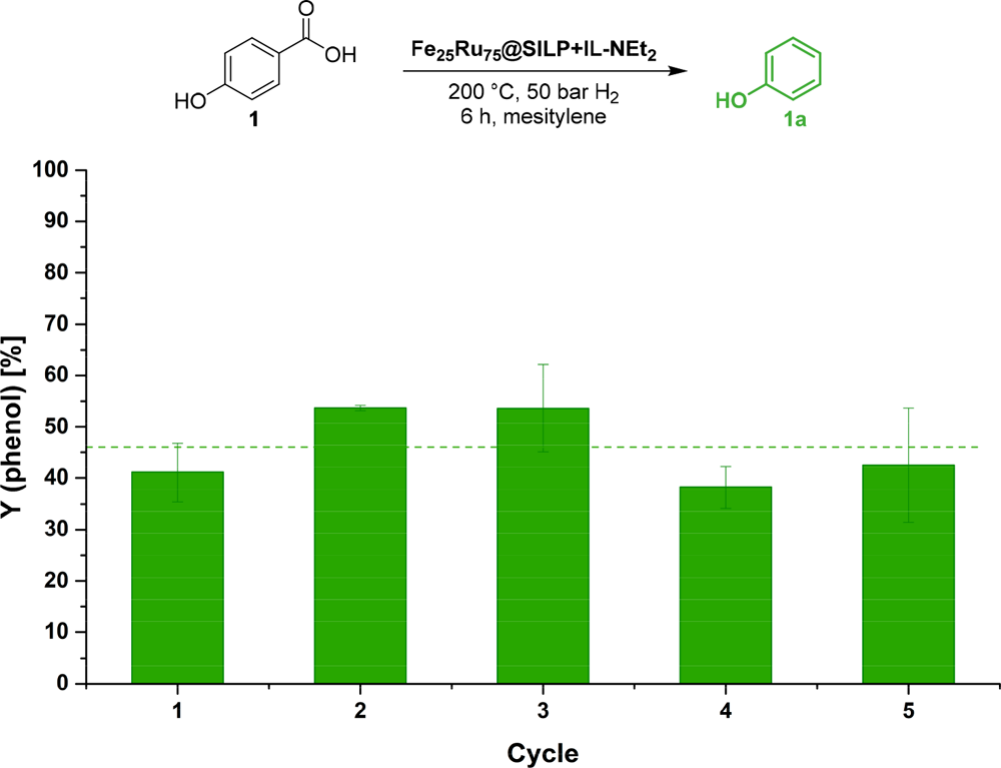
Catalyst recycling, decarboxylation of 4-hydroxybenzoic acid (**1**). Conditions: Fe_25_Ru_75_@SILP+IL-NEt_2_ (20 mg), substrate = 30.6 mg (30 equiv related to metal), 1 mL of mesitylene, 50 bar of H_2_, 200 °C, 6 h, 500 rpm. Product yields were determined by GD-FID using tetradecane as internal standard. Selectivity toward **1a** >99%. Error bars represent the standard deviation for three experiments.

Since leaching of physisorbed ionic liquids is a well-known issue in solution phase catalysis, the stability of Fe_25_Ru_75_@SILP+IL-NEt_2_ was carefully studied through recycling experiments using 4-hydroxybenzoic acid (**1**) as a model substrate (Figure 5). Between each cycle, a sample of the reaction mixture was taken, and the catalyst was washed with mesitylene. The reaction time was set to 6 h to ensure an incomplete conversion of the substrate and allow observing any change in activity or selectivity upon recycling. The yield associated with the formation of phenol was fairly constant (48% on average) for at least five cycles without any makeup or regeneration. The fluctuations observed are presumably due to the recycling procedure adopted, during which traces of products may have stayed on the catalyst between each cycle (see the Supporting Information for the detailed recycling procedure). Characterization of the catalyst with quantitative SEM-EDS after five cycles did not evidence significant variations in the total metal loading or in the Fe:Ru ratio, indicating the absence of leaching of the metals (Table S8). TEM analysis showed no noticeable growth nor aggregation of the NPs. In future studies, potential changes in the NPs’ bimetallic structure and electronic properties will be investigated by *ex situ* and operando X-ray absorption spectroscopy and magnetic measurements. N_2_ adsorption experiments indicated that the textural properties (BET surface area, pore size, and pore volume) of Fe_25_Ru_75_@SILP+IL-NEt_2_ were conserved. Titration of the available amine functionalities evidenced a similar loading as that on the fresh catalyst, confirming the absence of leaching of the IL-NEt_2_ under reaction conditions (Table S8). The analytical data indicate no sign of amidation of the carboxylic acid.

## Conclusion

In conclusion, the catalytic decarboxylation and tandem reduction/decarboxylation of hydroxy- and aminobenzoic acid derivatives were established as a versatile synthetic pathway for the synthesis of substituted phenols and anilines. A molecular approach was used to assemble bimetallic FeRu NPs and basic amine functionalities on a single support to produce a multifunctional Fe_25_Ru_75_@SILP+IL-NEt_2_ catalytic system. Operating under a H_2_ atmosphere was found to be essential for high catalytic activity, and labeling experiments showed that protons from the gas phase are incorporated in the products during decarboxylation. This highlights the synergistic action of the amine functionalities and FeRu NPs, the first being responsible for the activation of the carboxylic acid functionality, while the second presumably assists the protonolysis for product release through H_2_ activation. Consequently, the hydrogen activation and proton transfer were exploited by integrating the decarboxylation with selective hydrogenation and hydrodeoxygenation of substituted substrates. The novel tandem reaction sequence expands significantly the scope of hydroxybenzoic acids that can be converted and thus the variety of accessible phenolic structures. This approach provides a promising alternative strategy for the synthesis of valuable phenol derivatives from readily available hydroxybenzoic acids including substrates that can be derived from biomass feedstock or through biosynthesis.

## Supporting Information Available

Supplementary Tables and Figures, and Experimental Section describing catalysts synthesis, characterization methods, and protocols for catalytic experiments (PDF)
