# Supplementary material for: Decarboxylation and Tandem Reduction/Decarboxylation Pathways to Substituted Phenols from Aromatic Carboxylic Acids Using Bimetallic Nanoparticles on Supported Ionic Liquid Phases as Multifunctional Catalysts
Source: J Am Chem Soc. 2023 Oct 10;145(41):22845–54. doi: 10.1021/jacs.3c09290 (PMC10591467; doi:10.1021/jacs.3c09290)
Supplement: Supplementary file 1 — ja3c09290_si_001.pdf [file ja3c09290_si_001.pdf]

Supporting Information

Decarboxylation and Tandem Reduction/Decarboxylation Pathways to Substituted Phenols from Aromatic Carboxylic Acids using Bimetallic Nanoparticles on Supported Ionic Liquid Phases as Multifunctional Catalysts

Natalia Levin,[a]‡ Lisa Goclik,[ab]‡ Henrik Walschus,[a] Neha Antil,[a] Alexis Bordet,[a]* Walter Leitner[ab]*

[a] Max-Planck Institute for Chemical Energy Conversion, Stiftstr. 34-36, 45470 Mülheim an der Ruhr, Germany. E-Mail: alexis.bordet@cec.mpg.de, walter.leitner@cec.mpg.de

[b] Institut für Technische und Makromolekulare Chemie, RWTH Aachen University, Worringerweg 2, 52074 Aachen, Germany.

‡ Authors contribute equally

Table of Contents

1. General 2
2. Analytics 2
3. GC-Analysis 2
4. Synthesis of $Fe_{25}Ru_{75}$@SILP+IL-NR_2 3
5. Titration of IL-NEt_2 9
6. DFT calculations 9
7. Autoclave Reactions 11
8. Catalyst Recycling 11
9. Isolation of Products 12
10. Experiments with D_2 12
11. Supplementary Figures and Tables 13
11. References 28

Safety Warning

High pressure experiments with compressed gases must be carried out only with appropriate equipment and under rigorous safety precautions.

1. General

If not otherwise stated, the synthesis of ionic liquids (ILs), supported ionic liquid phases (SILPs) and nanoparticles immobilized on SILPs (NPs@SILPs) were carried out under an inert atmosphere (Ar) using standard Schlenk techniques or inside a glovebox. All synthesized materials were stored under inert atmosphere. All other chemicals and solvents were commercially available and used without further purification.

2. Analytics

All solution state NMR were recorded on a Bruker Ascend 400 spectrometer at room temperature. The coupling constants (*J*) are given in Hertz (Hz), and the chemical shifts (*δ*) are expressed in ppm, relative to TMS at 25 °C. Gas chromatography (GC) was performed on a Shimadzu GC-2030 equipped with a FID-detector and a CP-WAX-52CB column from Agilent. Gas chromatography coupled with a mass spectrometer (GC-MS) were performed on a Shimadzu QP2020. N_2 adsorption experiments were performed on a Quadrasorb SI from Quantachrom Instruments. Transmission and scanning transmission electron microscopy (TEM and STEM) were performed on a Hitachi HF2000 cold FEG operating at 200 kV at the Max-Planck-Institut für Kohlenforschung. Samples were prepared by depositing the powder onto a copper TEM grid with an amorphous carbon support film. Scanning electron microscopy with energy dispersive X-ray spectroscopy (SEM/EDS) was performed on a Hitachi S-3500N operating at 30kV at the Max-Planck-Institut für Kohlenforschung. Samples were prepared by depositing the powder onto a tape and flattening it out to obtain an even surface for analysis.

3. GC-Analysis

Yields were determined by gas chromatography (Shimadzu Nexis 2030) equipped with a FID detector using the methods described in the table below. Method 1 was used for substrate **1** and **17**. Method 2 was used for substrate **9**. Method 3 was used for substrates **6** and **12**. Method 4 was used for substrate **16** and method 5 was used for substrates **1-5, 7, 8, 10, 11, 13-15**.

Methods for GC analysis of the liquid phase.

Method Nr.	1	2	3	4	5
**Stationary phase (Column)**	Rtx-1701 (0.25 µm, 0.25 mm, 30 m)	Rtx-1 (0.5 µm, 0.25 mm, 30 m)	Rtx-5 (1.0 µm, 0.25 mm, 30 m)	Rtx-1 (0.5 µm, 0.25 mm, 30 m)	Rtx-1 (0.5 µm, 0.25 mm, 30 m)
**Mobile Phase [mL/min] (Carrier Gas)**	1.9 (He)	1.22 (He)	1.71 (He)	1.25 (He)	1.21 (He)
**Flow Control Mode Linear Velocity [cm/sec]**	40	30	40	30	30
**Injection Volume [µL]**	0.5	0.2	0.6	0.6	0.2
**Injection Temperature [°C]**	270	250	320	250	250
**Split Ratio**	25	30	30	35	30
**Temperature Program [°C]**	50 for 5 min, to 270 with 20 °C/min, 270 for 9 min	60 for 12 min, to 200 with 12 °C/min, 200 for 8 min	100 for 1 min, to 320 with 10 °C/min, 320 for 2 min	50 for 8 min, to 200 with 15 °C/min, to 300 with 25 °C/min, 300 for 22 min	65 for 10 min, to 200 with 15 °C/min, 200 for 8 min
**Detector Temperature [°C]**	275	260	330	310	260

Method for GC analysis of the gas phase.

Method Nr.	6
**Stationary phase (Column)**	Carboxen 1010 Plot (15 µm, 0.32 mm, 30 m)
**Mobile Phase [mL/min] (Carrier Gas)**	1.58 (He)
**Flow Control Mode Linear Velocity [cm/sec]**	30
**Injection Volume [µL]**	400
**Injection Temperature [°C]**	230
**Split Ratio**	50
**Temperature Program [°C]**	35 for 6 min, to 150 with 15°C/min, 150 for 9 min
**Detector Temperature [°C]**	230

The identification of the compounds was done by injecting the pure compounds in GC-FID, or by combination with GC-MS.

4. Synthesis of $Fe_{25}Ru_{75}$@SILP+IL-NR_2

4.1. Synthesis of IL-NEt_2

4.1.1. Synthesis of 1-[2-(diethylamino)ethyl]-3-butylimidazolium bromide hydrobromide

Following a published procedure,[1] 2-Bromo-N,N-diethylethylamine hydrobromide (7.26 g, 27.8 mmol) was suspended in 100 mL dry acetonitrile and heated at 80 °C until complete dissolution, giving a light yellow solution. Under Ar, 1-butylimidazole (3.6 mL, 29 mmol) was added slowly to the mixture under stirring and the reaction medium was refluxed at 150 °C for 48 h. After this period, the reaction was left to cool down to room temperature, the solvent was evaporated and the resulting yellow paste was washed with dry acetone (4 x 10 mL) and dry diethylether (4 x 10 mL). The remaining solid was dried under vacuum for 3 h at room temperature and 1-[2-(diethylamino)ethyl]-3-butylimidazolium bromide hydrobromide was yielded as white solid (6.27 g, 20.60 mmol, 74%).

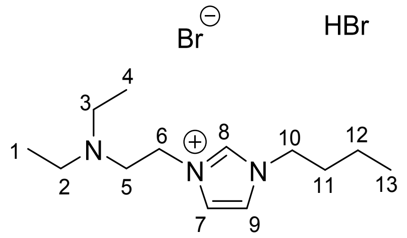

^{1}H-NMR (400 MHz, DMSO-d_6): δ (ppm) =9.80 (s, 1H, *H*Br), 9.41 (s, 1H, *H8*), 7.95 (s, 1H, *H9*), 7.86 (s, 1H, *H7*), 4.66 (t, *J* = 6.8 Hz, 2H, *H6*), 4.19 (t, *J* = 7.2 Hz, 2H, *H10*), 3.66 (t, *J* = 7.0 Hz, 2H, *H5*), 3.23 (q, *J* = 7.3 Hz, 4H, *H2*, *H3*), 1.84-1.77 (m, 2H, *H11*), 1.35-1.23 (m, 8H, *H1*, *H4*, *H12*), 0.92 (t, *J* = 7.3 Hz, 3H, *H13*).

^{13}C-NMR (400 MHz, DMSO-d_6): δ (ppm) =137.42 (1C, *C8*), 123.13 (1C, *C9*), 123.06 (1C, *C7*), 49.88 (1C, *C5*), 49.21 (1C, *C6*), 47.43 (2C, *C2*, *C3*), 43.61 (1C, *C10*), 31.34 (1C, *C11*), 19.29 (1C, *C12*), 13.80 (1C, *C13*), 8.86 (2C, *C1*, *C4*).

4.1.2. Synthesis of 1-[2-(diethylamino)ethyl]-3-butylimidazolium bis(trifluoromethane) sulfonamide

1-[2-(diethylamino)ethyl]-3-butylimidazolium bromide hydrobromide (10.94 g, 28.41 mmol) was dissolved in ethanol (60 mL) under an inert atmosphere. Under Ar, pyridine (2.4 mL, 2.41 g, 30.51 mmol, 1.07 equiv.) was added to the mixture, along with additional 10 mL of ethanol to rinse the Schlenk flask, after which the reaction medium was stirred for 10 min at room temperature. Finally, $LiNTf_2$ (8.64 g, 30.09 mmol) was added to the solution, along with additional 10 mL of

ethanol to rinse, and the resulting solution was stirred under Ar at room temperature for 15 h. Afterwards, the solvent was evaporated and the resulting pale orange residue was dissolved in DCM (40 mL). The solution was filtered through celite (previously washed with 2 x 10 mL DCM). Additional 20 mL of DCM were added to rinse the flask. Due to the remaining of a small amount of a whitish thin solid in suspension, the solution was filtered through celite a second time, giving a clear light-yellow solution. Finally, the solvent was removed from the recovered solution. The product was dissolved and stirred for 90 min in a mixture of H_2O (30 mL) and DCM (60 mL) to remove any remaining trace of pyridine. The organic layer was washed with 3 x 20 mL H_2O, dried with anhydrous Na_2SO_4 and finally the solvent was removed under vacuum to afford a thick light-yellow oil that was dried under vacuum overnight at room temperature. 1-[2-(diethylamino)ethyl]-3-butylimidazolium bis(trifluoromethane) sulphonamide was obtained as a viscous liquid (7.95 g, 15.76 mmol, 77%).

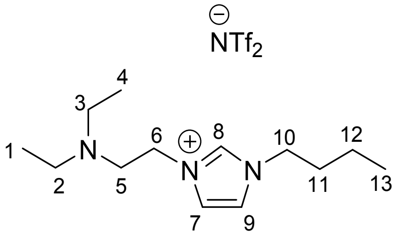

^{1}H-NMR (400 MHz, DMSO-d_6): δ (ppm) = 9.17 (s, 1H, *H8*), 7.80 (d, J = 8.9 Hz, 1H, *H7, H9*), 4.43 (t, J = 6.4 Hz, 2H, *H6*), 4.20 (t, J = 7.2 Hz, 2H, *H10*), 3.26 (t, J = 6.1 Hz, 2H, *H5*), 2.92 (q, 4H, *H2*, *H3*), 1.83-1.75 (m, 2H, *H11*), 1.34-1.24 (m, 2H, *H12*), 1.08 (t, J = 7.2 Hz, 6H, *H1*, *H4*), 0.92 (t, J = 7.4 Hz, 3H, *H13*).

^{13}C-NMR (400 MHz, DMSO-d_6): δ (ppm) = 136.68 (1C, *C8*), 122.79 (1C, *C9*), 122.41 (1C, *C7*), 50.41 (1C, *C5*), 48.72 (1C, *C6*), 46.82 (2C, *C2*, *C3*), 44.99 (1C, *C10*), 31.40 (1C, *C11*), 18.78 (1C, *C12*), 13.17 (1C, *C13*), 9.70 (2C, *C1*, *C4*).

ESI-MS (+): m/z = 224.2, calculated for $[C_{13}H_{26}N_3]^+$ = 224.21. APCI-MS (-): m/z = 279.9, calculated for $[C_2F_6NO_4S_2]^-$ = 279.92; m/z = 566.9, calculated for $[LiNTf_2+NTf_2]^-$, $[C_4F_{12}LiN_2O_8S_4]^-$ = 566.85.

4.1.3. Synthesis of 1-[2-(diethylamino)ethyl]-3-butylimidazolium bromide

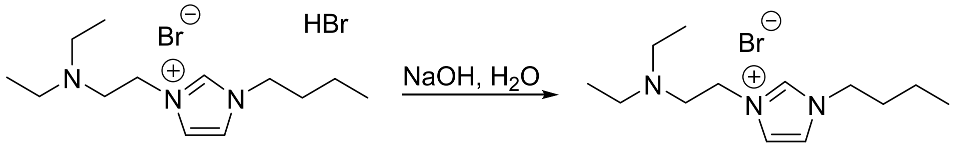

1-[2-(diethylamino)ethyl]-3-butylimidazolium bromide hydrobromide (3.8 g, 9.86 mmol) was dissolved in 2 mL of water, followed by addition of 1.2 equivalent of NaOH (0.47 g, 11.83 mmol) dissolved in 4 mL of water. The reaction mixture was stirred at room temperature for 15 minutes. After stirring, the water was removed under vacuum. To the resulting light yellow solid, DCM was added to dissolve the 1-[2-(diethylamino)ethyl]-3-butylimidazolium bromide and the solution was filtered. The solid was washed several times with DCM to extract the product in DCM, followed by the removal of DCM under vacuum. The remaining yellow liquid was dried under vacuum for 3 h at room temperature and 1-[2-(diethylamino)ethyl]-3-butylimidazolium bromide was yielded as viscous yellow liquid (2.8 g, 9.2 mmol, 93%).

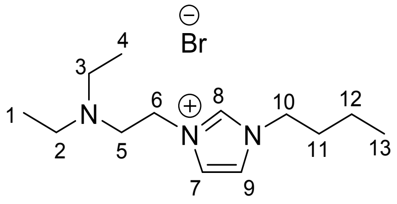

^{1}H NMR (400 MHz, DMSO-d_6): *δ* (ppm) = 9.24 (s, 1H, *H8*), 7.81 (d, *J* = 1.5 Hz, 2H, *H7, H9*), 4.21 (m, 4H, *H6, H10*), 2.73 – 2.67 (m, 2H, *H5*), 2.43 (q, *J* = 7.1 Hz, 4H, *H2, H3*), 1.81 – 1.69 (m, 2H, *H11*), 1.23-1.13 (m, 2H, *H12*), 0.88 (t, *J* = 7.4 Hz, 3H, *H13*), 0.82 (t, *J* = 7.1 Hz, 6H, *H1, H4*).

^{13}C NMR (101 MHz, DMSO): *δ* (ppm) = 136.86 (1C, *C8*), 123.25 (1C, *C9*), 122.41 (1C, *C7*), 52.33 (1C, *C5*), 48.82 (1C, C6), 47.89 (1C, *C10*), 46.71 (2C, *C2*, *C3*), 31.94 (1C, *C11*), 19.12 (1C, *C12*), 13.70 (1C, *C13*), 12.26 (2C, *C1*, *C4*).

ESI-MS (+): m/z = 224.2, calculated for $[C_{13}H_{26}N_3]^+$ = 224.21. ESI-MS (-): m/z = 79, calculated for $[Br]^-$ = 78.92.

4.2. Synthesis of IL-NH_2

4.2.1. Synthesis of 1-[2-(ethylamino)]-3-butylimidazolium bromide hydrobromide

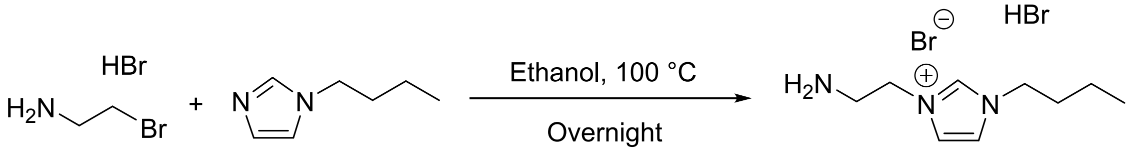

Following a published procedure [https://doi.org/10.1002/smll.202206806], 2-bromoethylamine hydrobromide (4 g, 19.5 mmol, 1.1 eq) was first dried under vacuum at 80 °C overnight prior to use, and then weighed into a Schlenk flask in the glovebox, and dissolved in ethanol (30 mL,

anhydrous). 1-butylimidazole (2.13 mL, 17.2 mmol, 1 eq) was added slowly to the mixture under stirring. The mixture was then refluxed for 15 h at 110 °C under argon. After cooling down the reaction, ethanol was removed under vacuum at 50 °C in an oil bath, forming a thick light yellow liquid, which was washed with THF (3 x 20 mL, anhydrous) and diethyl ether (1 x 20 mL, anhydrous). The product was purified using a neutral alumina column and then dried overnight under vacuum at room temperature to give 1-[2-(ethylamino)]-3-butylimidazolium bromide hydrobromide as yellow liquid (2.4 g, 7.22 mmol, 42% yield).

4.2.2. Synthesis of 1-[2-(ethylamino)]-3-butylimidazolium bromide

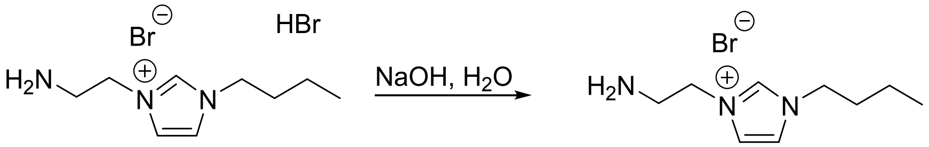

1-[2-(ethylamino)]-3-butylimidazolium bromide hydrobromide (2.0 g, 6.07 mmol) was dissolved in 2 mL of water, followed by addition of 1.2 equivalents of NaOH (0.28 g, 7.28 mmol) dissolved in 4 mL of water. The mixture was stirred at room temperature for 15 minutes. After stirring, the water was removed under vacuum. To the resulting light yellow solid, DCM was added to dissolve the 1-[2-(ethylamino)]-3-butylimidazolium bromide and the solution was filtered. The solid was washed several times with the DCM, to extract the product in DCM, followed by the removal of DCM under vacuum. The remaining yellow liquid was dried under vacuum for 3 h at room temperature and 1-[2-(ethylamino)]-3-butylimidazolium bromide was yielded as yellow liquid (1.2 g, 4.8 mmol, 80%).

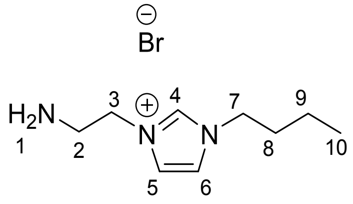

^{1}H NMR (400 MHz, DMSO-d_6): δ (ppm) = 9.27 (s, 1H, *H4*), 7.81 (d, *J* = 1.5 Hz, 2H, *H5, H6*), 4.22 – 4.16 (m, 4H, *H3, H7*), 3.32 (s, 2H, *H1*), 2.96 – 2.89 (m, 2H, *H2*), 1.81 – 1.72 (m, 2H, *H8*), 1.25 (m, 2H, *H9*), 0.88 (t, *J* = 7.4 Hz, 3H, *H10*).

^{13}C NMR (101 MHz, DMSO): δ (ppm) = 136.86 (1C, *C4*), 123.18 (1C, *C6*), 122.68 (1C, *C5*), 51.79 (1C, *C3*), 48.98 (1C, C7), 41.41 (1C, *C2*), 31.83 (1C, *C8*), 19.32 (1C, *C9*), 13.83 (1C, *C10*).

ESI-MS (+): m/z = 168.1, calculated for $[C_9H_{18}N_3]^+$ = 168.15. ESI-MS (-): m/z = 79, calculated for $[Br]^-$ = 78.92.

4.2.3. Synthesis of 1-[2-(ethylamino)]-3-butylimidazolium bis(trifluoromethane) sulfonamide

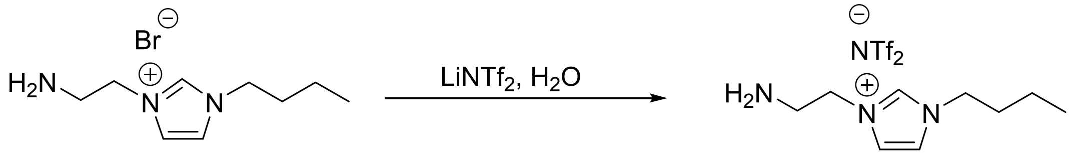

1-[2-(ethylamino)]-3-butylimidazolium bromide (0.210 g, 0.88 mmol) was dissolved in 2 mL of water. Then, $LiNTf_2$ (0.268 g, 0.93 mmol) was added to the solution followed by the stirring of reaction mixture at room temperature for 48 h. After stirring, the water was removed under vacuum. To the resulting yellow liquid, DCM was added to dissolve the 1-[2-(ethylamino)]-3-butylimidazolium bis(trifluoromethane) sulphonamide, and the solution was filtered, followed by the removal of DCM under vacuum. 1-[2-(ethylamino)]-3-butylimidazolium bis(trifluoromethane) sulphonamide was yielded as yellow liquid (0.260 g, 60 mmol, 68%)

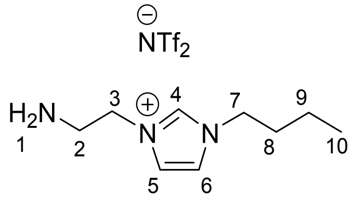

^{1}H NMR (400 MHz, DMSO-d_6): δ (ppm) = 8.68 (s, 1H, *H4*), 7.42 (s, 1H,*H6*), 7.28 (s, 1H, *H5*), 4.27 (t, *J* = 6.0 Hz, 2H, *H3*), 4.20 – 4.14 (m, 2H, *H7*), 3.26 – 3.15 (m, 2H, *H2*), 3.19 (s, 2H, *H1*), 1.92 – 1.80 (m, 2H, *H8*), 1.38 (m, 2H, *H9*), 0.97 (t, *J* = 7.4 Hz, 3H, *H10*).

^{13}C NMR (101 MHz, DMSO): δ (ppm) = 135.84 (1C, *C4*), 123.24 (1C, *C6*), 122.64 (1C, *C5*), 51.87 (1C, *C3*), 50.53 (1C, C7), 41.12 (1C, *C2*), 32.10 (1C, *C8*), 19.76 (1C, *C9*), 13.38 (1C, *C10*).

4.3. Synthesis of $Fe_{25}Ru_{75}$@SILP

A solution of $\{Fe[N(Si(CH_3)_3)_2]_2\}_2$ (18.0 mg, 0.05 mmol Fe) in mesitylene (2 mL) was combined with a solution of [Ru(cod)(cot)] (47.0 mg, 0.15 mmol Ru) in mesitylene (2 mL) in a Fischer-Porter bottle (70 mL). The SILP (500 mg) was added to the solution of the metal precursors along with mesitylene (1 mL) and the reaction mixture was stirred under argon at room temperature for 30 min. The Fischer-Porter bottle was evacuated and backfilled with H_2 and the suspension was stirred under H_2 (3 bar) at 150 °C for 18 h. Under this reducing environment a black powder was

obtained, indicating the immobilization of the NPs onto the SILP. Mesitylene was decanted and $Fe_{25}Ru_{75}$@SILP were washed with fresh toluene (3 x 3 mL) and dried *in vacuo* at room temperature for 1 h.

4.4. Synthesis of $Fe_{25}Ru_{75}$@SILP+IL-NEt_2.

The physisorption of IL-NEt_2 onto $Fe_{25}Ru_{75}$@SILP was achieved following an adapted procedure from literature, replacing IL-SO_3H by IL-NEt_2.[2] In this case, a suspension of $Fe_{25}Ru_{75}$@SILP (187.9 mg, 0.0752 mmol) was prepared in an acetone solution (3 mL) of IL-NEt_2 (109.6 mg, 0.217 mmol, 2.89 equiv.). The mixture, prepared in a glove box, was stirred for 1 h at room temperature, after which it was taken out of the glove box and the solvent was evaporated in a Schlenk line. Finally, the catalyst was dried under vacuum for 2 h at room temperature.

A similar procedure was followed to prepare $Fe_{25}Ru_{75}$@SILP+IL-NHEt and $Fe_{25}Ru_{75}$@SILP+IL-NH_2.

5. Titration of IL-NEt_2

For the titration of the IL-NEt2, the species with Br- as anion was selected for its good solubility in water. 150 mg of IL-NEt2(Br) were dissolved in 10 mL of MilliQ water and were titrated with a 0.1 M HCl solution (small aliquots added with Eppendorf pipettes). The pH of the solution was followed during the whole titration with a Mettler Toledo Seven Excellence Multiparameter pH-meter equipped with a MTPH Sensor Science Pro-ISM. This allowed for the titration curve to be plotted and the equivalence volume to be determined thanks to the minimum of its second derivative. The pH corresponding to a volume equal to one half of the equivalence point allows determination of the pKaH.

6. DFT calculations

6.1. Computational details

All quantum chemical calculations were performed with the ORCA quantum chemistry software package (Version 5.0.4).[3] For all species studied here, the calculations employed density functional theory (DFT) with the B3LYP hybrid functional[4-6] and the def2-TZVP basis set.[7] The conductor-like polarizable continuum model (CPCM)[8] was used for charge compensation in all calculations, employing different solvent models for each case. Each species was optimized directly with the corresponding CPCM model, after which a frequency calculation was performed to ensure that a minimal stationary point was reached. Likewise, after the frequency calculation the free Gibbs energy of the species was obtained and was used for further calculations.

6.2. Thermodynamics of the decarboxylation reaction

The thermodynamics of the decarboxylation reaction of different benzoic acids to their decarboxylation product and CO_2 was investigated with a CPCM model of heptane, considering a dielectric constant of 1.923.[9] The reaction considered is shown below, and the ΔG of the reaction was calculated employing equation S1.

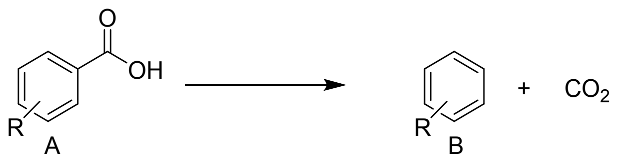

$\Delta G^0_{total} = G^0_{CO_2} + G^0_B - G^0_A$ (eq. S1)

The calculated values for and their comparison to reported values are shown in Table S1.

6.3. Calculations of pK_{aH} of amines in water

To express the basicity of a base B, the pK_{aH} is used to refer to its protonation, according to equations S2 and S3.[10]

$BH^+ + S \rightleftarrows B + SH^+$ (eq. S2)

$pK_{aH} = -log\frac{a[B].a[SH^+]}{a[BH^+]}$ (eq. S3)

where S is the solvent and a the activity of each species in solution.

In order to allow for cancellation of some errors that appear while calculating the basicity of a base, the isodesmic method is used to calculate a relative pK_{aH}, according to a previously published procedure.[11-13] Following this method, the pK_{aH} of the base B can be calculated with the following equation S4.

$pK_{aH} = \frac{\Delta G}{RT\ln(10)} + pK_{aH,\ ref}$ (eq. S4)

where p$K_{aH,\ ref}$ is the known pK_{aH} value of a base taken as reference and ΔG refers to the free Gibbs energy of equation S5 and is calculated with equation S6.

$BH^+ + B_{ref} \rightleftarrows B + BH^+_{ref}$ (eq. S5)

$\Delta G = G_B + G_{BH^+_{ref}} - G_{BH^+} - G_{B_{ref}}$ (eq. S6)

In our investigation, we used this procedure to calculate the pK_{aH} values of different bases taking as a reference pyridine, since its pK_{aH} value in organic solvent is well known (5.23 in water) and it is usually employed to reference pK_{aH} values of other bases.[10] To calculate ΔG from equation S6 we turned to quantum chemical calculations as explained in section 5.1. The different species were optimized with a CPCM model of the solvent as implemented in ORCA 5.0.4. and through a frequency calculation the free Gibbs energy of the species was obtained and used in equation S6.

The results obtained and their comparison to experimental are shown in table S2.

7. Autoclave Reactions

As example, the autoclave reaction procedure for the decarboxylation of 4-hydroxybenzoic acid (**1**) is described. For other substrates, the amounts of substrate, catalyst or solvent and the reaction conditions can be varied. However, the overall procedure stays the same.

The substrate (65 eq. with respect to metal, 0.221 mmol), catalyst (10 mg, containing 0.0034 mmol metal) and solvent (heptane, 0.5 mL) were weighed inside of a glovebox into the autoclave. The autoclave was closed, transferred out of the glovebox and pressurized with 50 bar hydrogen. After heating the autoclave under stirring (500 rpm) to 175 °C for 18 h, it was cooled down in a water bath and depressurized. The GC samples were prepared by adding acetone (250 mg) to the reaction mixture and taking a sample through a syringe with a filter. To the sample, tetradecane (internal standard, 20 mg) and acetone (200 mg) were added. For all amines, methanol/dioxane (1/1) was used as solvent for the preparation of GC samples.

8. Catalyst Recycling

The substrate (33 eq. with respect to metal, 0.11 mmol), catalyst (20 mg, containing 0.0068 mmol metal) and solvent (mesitylene, 0.5 mL) were weighed inside of a glovebox into the autoclave. The autoclave was closed, transferred out of the glovebox and pressurized with 50 bar hydrogen. After heating the autoclave under stirring (500 rpm) to 200 °C for 6 h, it was cooled down in a water bath and depressurized. The glass insert was taken out and transferred to a Schlenk tube. The tube was evaporated carefully until the first bubbles from the evaporating solvent show up. Then it was backfilled with argon. This procedure was performed five times and the Schlenk tube with the reaction mixture was transferred into the glovebox. Mesitylene (1 mL) was added to the reaction mixture to solute the product. The supernatant was taken out with a syringe to prepare a GC sample and the catalyst was washed three times with fresh mesitylene. Since the substrate is not soluble in mesitylene, it remained in the autoclave vial. To the washed catalyst were added

new substrate (the amount, which was converted in the cycle before) and solvent. The autoclave was closed, pressurized and heated like before to start the new cycle.

Tetradecane (internal standard, 20 mg) and acetone (200 mg) were added to the recovered supernatant to prepare the GC sample.

9. Products Isolation

The isolation of the products was carried out by filtration of the reaction mixture through a syringe filter and purification via silica column (EtOAc:Cyclohexane = 1:10). After careful evaporation of the solvent, the pure compounds were yielded and characterized by ^{1}H and ^{13}C NMR.

10. Experiments with D_2

4-Hydroxybenzoic acid (61.1 mg, 65 eq. with respect to metal, 0.442 mmol), $Fe_{25}Ru_{75}$@SILP+IL-NEt_2 (20 mg, containing 0.007 mmol metal) and solvent (heptane, 1.0 mL) were weighed inside of a glovebox into the autoclave. The autoclave was closed, transferred out of the glovebox and pressurized with 20 bar deuterium. After heating the autoclave under stirring (500 rpm) to 200 °C for 18 h, it was cooled down in a water bath and depressurized. The isolation of the product was carried out by purification via silica column (EtOAc:Cyclohexan = 1:10). After careful evaporation of the solvent, the pure compound was yielded. The product was analyzed by GC-MS and NMR.

As reference experiment, phenol was used as substrate under the same reaction conditions and the product was isolated and analyzed accordingly.

Comparing the ^{1}H (Figure 2) and ^{13}C NMR of the isolated products to the NMR of phenol showed a different excess of deuteration of the products (Table S6). Comparing the ^{1}H and ^{13}C NMR of this isolated product to that of non-deuterated phenol evidenced the nearly complete deuteration of the positions 2, 4, and 6 of the aromatic ring. This deuteration caused a triplet shape of the carbon peaks of the carbons in position 2, 4 and 6 in $^{13}C\{^{1}H\}$ NMR. In contrast, exposing non-deuterated phenol to the same reaction conditions led to a phenol with deuteration only on positions 2 and 6, as expected in alpha of the –OH group. Here, the signal for the carbon in position 4 in $^{13}C\{^{1}H\}$ NMR is a singlet. These results show that a proton from the gas phase replaces the carboxylic acid functionality during decarboxylation and evidence the importance of having an H_2 atmosphere to observe high decarboxylation activity. This also highlights the synergistic action of the amine functionalities and FeRu NPs, the first being responsible for the activation of the carboxylic acid functionality while the second presumably accelerates the cleavage reaction by activating H_2, thus providing protons and facilitating the protonolysis step.

11. Supplementary Tables and Figures

Table S1: Calculated ΔG values of decarboxylation reactions.

Decarboxylation of:	ΔG / kcal.mol^{-1}	Solvent	Functional + basis set	Software	Reference
**Benzoic acid**	-15.9	PCM DMSO	B3PW91 6-31G*	Gaussian 03	[14]
**Benzoic acid**	-17.4	CPCM DMSO	B3LYP def2-TZVP	Orca 5.0.4	This work
***o*-chlorobenzoic acid**	-19.5	COSMO DMSO	BP TZP	ADF2009	[15]
***o*-chlorobenzoic acid**	-23.9	CPCM DMSO	B3LYP def2-TZVP	Orca 504	This work
**4-hydroxybenzoic acid**	-17.7	CPCM Heptane	B3LYP def2-TZVP	Orca 5.0.4	This work

All the calculations were performed considering a temperature of 298.15 K.

Table S2: Calculated values of pK_{aH} in water and their comparison to experimental values.

pK_{aH} of:	Experimental	Calculated
IL-NEt_2	7.21 ± 0.01	7.3
IL-NH_2	-	4.8
NEt_3	10.7[a]	11.6
NEt_2H	11.0[a]	11.4
Butylamine	10.6[a]	10.3
N,N-dimethylaniline	5.1[a]	3.3
NPh_2H	0.8[a]	-3.5

All the calculations were performed considering a temperature of 298.15 K and a CPCM model of water. [a] Reported values from literature.[14]

Table S3: Variations of physisorbed ionic liquid loading.

	$Fe_{25}Ru_{75}$@SILP+IL-NEt_2 A	$Fe_{25}Ru_{75}$@SILP+IL-NEt_2 B	$Fe_{25}Ru_{75}$@SILP+IL-NEt_2 C
**IL-NEt_2 loading [mmol·g^{-1}] [a]**	0.44	0.731 ± 0.001[b]	1.07
**IL-NEt_2/metal ratio[a]**	1.4	2.9 ± 0.01[b]	5.8
**Accessible amines [mmol·g^{-1}]**	0.39 ± 0.001[c]	0.49 ± 0.004[b]	0.59 ± 0.004[c]
**Accessible amines [%]**	89.3 ± 0.02[c]	66.7 ± 0.6[b]	55.3 ± 0.4[c]
**X [%], Y_{1a}[%][d]**	32[e]	>99[f]	83[g]

[a] Determined based on the amount of IL-NEt_2 recovered after the physisorption procedure. [b] The average values and standard deviations were determined through the characterization of three batches of each material or [c] one batch of material measured in duplicate. [d] Conversion and yield for the decarboxylation of 4-hydroxybenzoic acid: catalyst (10 mg), solvent: heptane (0.5 mL), 175 °C, H_2 (50 bar), 18 h, 500 rpm with [e] 100 eq. substrate compared to metal and 30 eq. substrate compared to titrated amines, [f] 65 eq. substrate compared to metal and 30 eq. substrate compared to titrated amines, [g] 100 eq. substrate compared to metal and 70 eq. substrate compared to titrated amines,

Table S4: Catalysts characterization.

	$Fe_{25}Ru_{75}$@SILP	$Fe_{25}Ru_{75}$@SILP+IL-NEt_2
**IL-NEt_2 loading [mmol·g^{-1}]**		0.73[a]
**Accessible amines [mmol·g^{-1}]**	-	0.49 ± 0.004[b]
**BET surface area [m^2·g^{-1}]**	240 ± 1	36 ± 1
**Pore volume [m^3·g^{-1}]**	0.56 ± 0.01	0.13 ± 0.01
**Metal loading[b] [mmol·g^{-1}]**	0.40 ± 0.04	0.34 ± 0.03
**Metal ratio Fe : Ru[b] [%]**	23 ± 5 : 77 ± 5	27 ± 3 : 73 ± 4
**Particle size [nm]**	3.3 ± 0.6	3.1 ± 0.7

NP sizes were determined by TEM. Metal ratio and loading were determined using SEM-EDX (Fe-K / Ru-L). [a] Determined based on the amount of IL-NEt_2 recovered after the physisorption procedure. [b] The average values and standard deviations were determined through the characterization of three batches of each material.

Table S5: Decarboxylation of 4-hydroxybenzoic acid (**1**) using $Fe_{25}Ru_{75}$@SILP with different amines as additives.

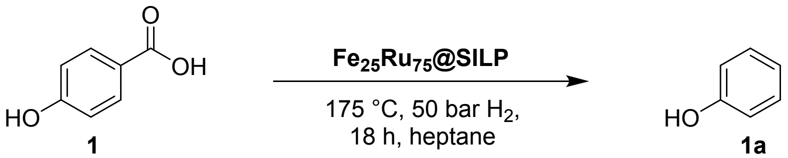

1 → **1a**

Additive	X [%]	Y_{1a} [%]	pK_{aH}
**Triethylamine**	>99	>99	10.7[a]
**Diethylamine**	>99	>99	11.0[a]
**Butylamine**	>99	>99	10.6[a]
**IL-NEt_2**	>99	>99	7.2[b]
**IL-NH_2**	6	6	4.8[c]
**N,N-Dimethylaniline**	61	61	5.1[a]
**Diphenylamine**	0	0	0.8[a]

Reaction conditions: Catalyst (metal content: 0.0034 mmol), additive (0.007 mmol), **1** (0.221 mmol, 65 eq. compared to metal, 30 eq. compared to amine), solvent: heptane (0.5 mL), 175 °C, H_2 (50 bar), 18 h, 500 rpm; X: Conversion, Y: Yield, determined by GD-FID using tetradecane as internal standard. [a] Literature values as reported in water.[10] [b] Determined experimentally in this study (Figure S1). [c] Calculated by DFT in this study.

Table S6: Deuterated positions of the products isolated from the decarboxylation of 4-hydroxybenzoic acid (**1**) using $Fe_{25}Ru_{75}$@SILP+IL-NEt_2 with application of D_2.

Entry	Substrate	Main product	Deuteration Position x[a] (Signal shape in ^{13}C NMR)				
			2	3	4	5	6
1	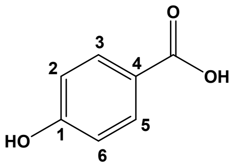	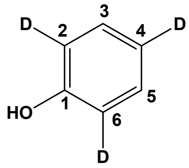	yes (t)	no	**yes (t)**	no	yes (t)
2	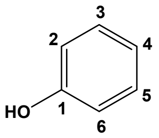	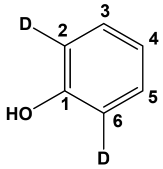	yes (t)	no	**no (s)**	no	yes (t)

[a] Determined by ^{1}H and ^{13}C{^{1}H} NMR of the isolated products; s = singlet, t = triplet. Reaction conditions: Catalyst (20 mg, metal content: 0.0068 mmol), substrate (0.442 mmol, 65 eq. with respect to metal), solvent: heptane (1 mL), 200 °C, D_2 (20 bar), 18 h, 500 rpm.

Table S7: Reference experiments for the decarboxylation of 2-hydroxy-5-octanoylbenzoic acid (**14**).

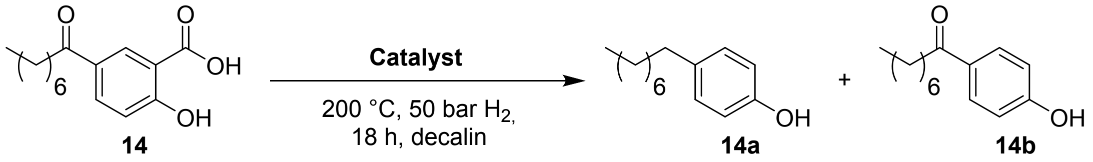

Catalyst	X [%]	Y_{14a} [%]	Y_{14b} [%]
**$Fe_{25}Ru_{75}$@SILP+IL-NEt_2**	>99	>99 (85)	0
**SILP+IL-NEt_2**	>99	0	>99

Reaction conditions: Catalyst (metal content: 0.0034 mmol), substrate (0.111 mmol, 33 eq. compared to metal, 15 eq. compared to amine), solvent: decaline (0.5 mL), 200 °C, H_2 (50 bar), 18 h, 500 rpm; X: Conversion, Y: Yield, determined by GC-FID using tetradecane as internal standard. Isolated yields in parenthesis.

Table S8: Characterization of $Fe_{25}Ru_{75}$@SILP+IL-NEt_2 before and after catalysis.

	Before catalysis	After catalysis (5 cycles)
**Accessible amines [mmol·g^{-1}]**	0.487 ± 0.004[a]	0.489 ± 0.007[b]
**Accessible amines [%]**	66.7 ± 0.6[a]	67 ± 1[b]
**Surface area [m^2·g^{-1}]**	36 ± 1	35 ± 1
**Pore volume [m^3·g^{-1}]**	0.13 ± 0.002	0.14 ± 0.001
**Metal loading[a] [mmol·g^{-1}]**	0.34 ± 0.02	0.32 ± 0.05
**Metal ratio Fe : Ru[a] [%]**	27 ± 3 : 73 ± 4	22 ± 7 : 78 ± 4
**Particle size [nm]**	3.1 ± 0.7	2.5 ± 0.5

NP sizes were determined by TEM. Metal ratio and loading were determined using SEM-EDX (Fe-K / Ru-L). The average values and standard deviations were determined through the characterization of [a] three batches of each material or [b] one batch of material measured in duplicate.

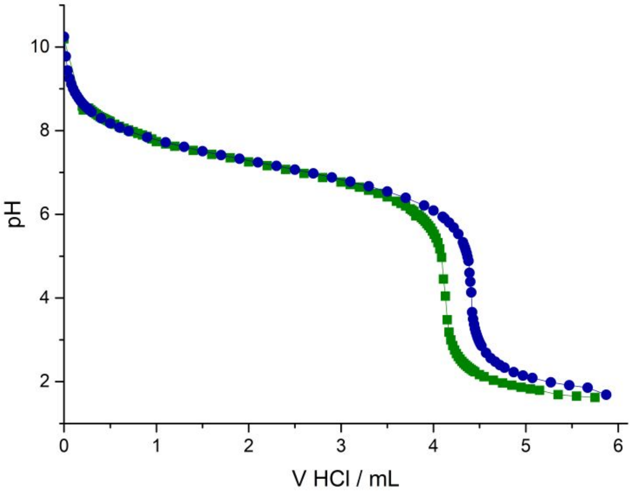

Figure S1: Titration curve of IL-NEt_2(Br) in water with HCl 0.1 M (duplicate). Average value: pK_{aH} = 7.21 ± 0.01.

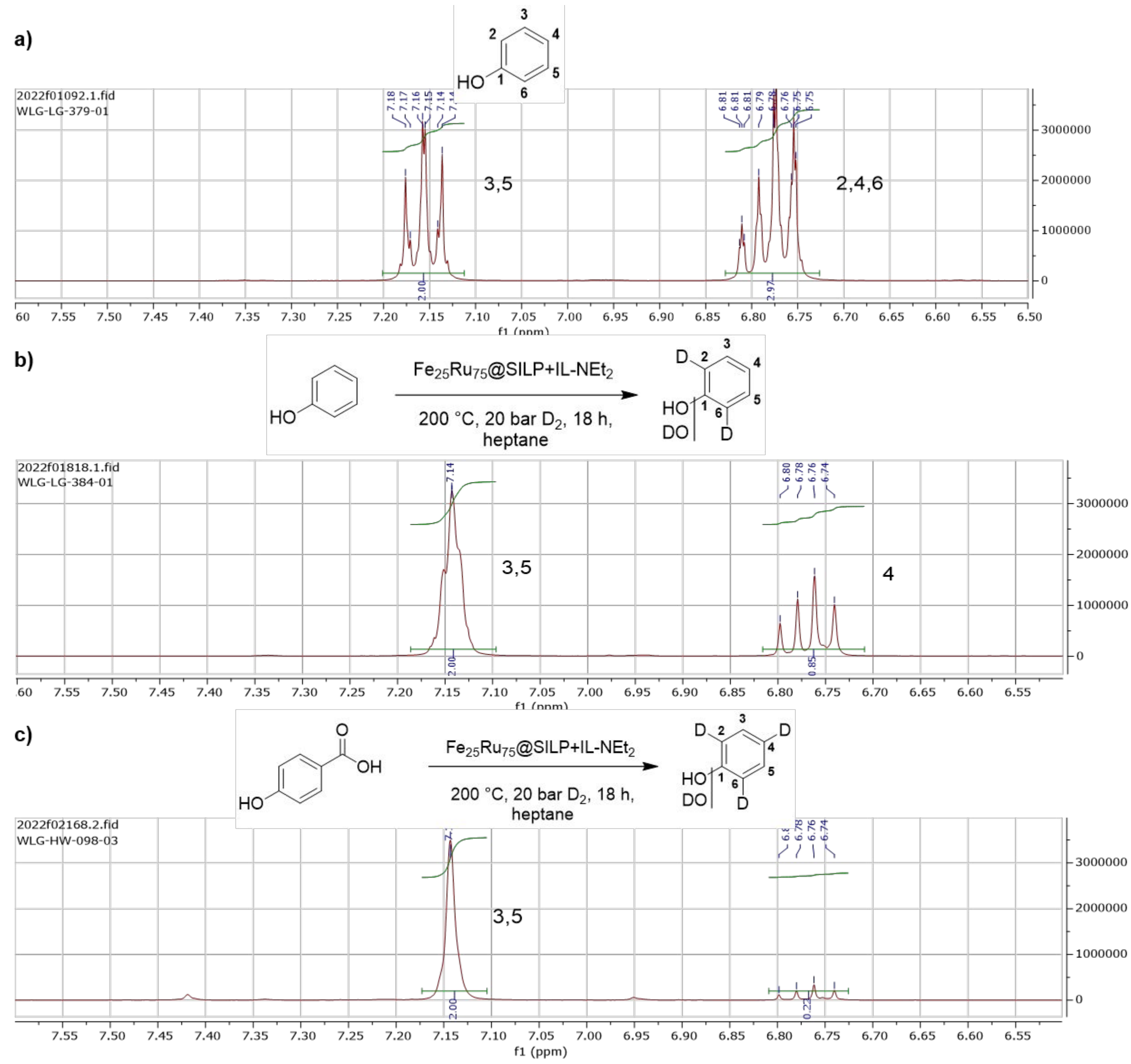

Figure S2: ^{1}H NMR (400 MHz, methanol-d_4) spectra of a) phenol, b) product, isolated from applying phenol to decarboxylation conditions under D_2 and c) product, isolated from decarboxylation of 4-hydroxybenzoic acid under D_2. For ^{13}C NMR spectra, see section 9.1.

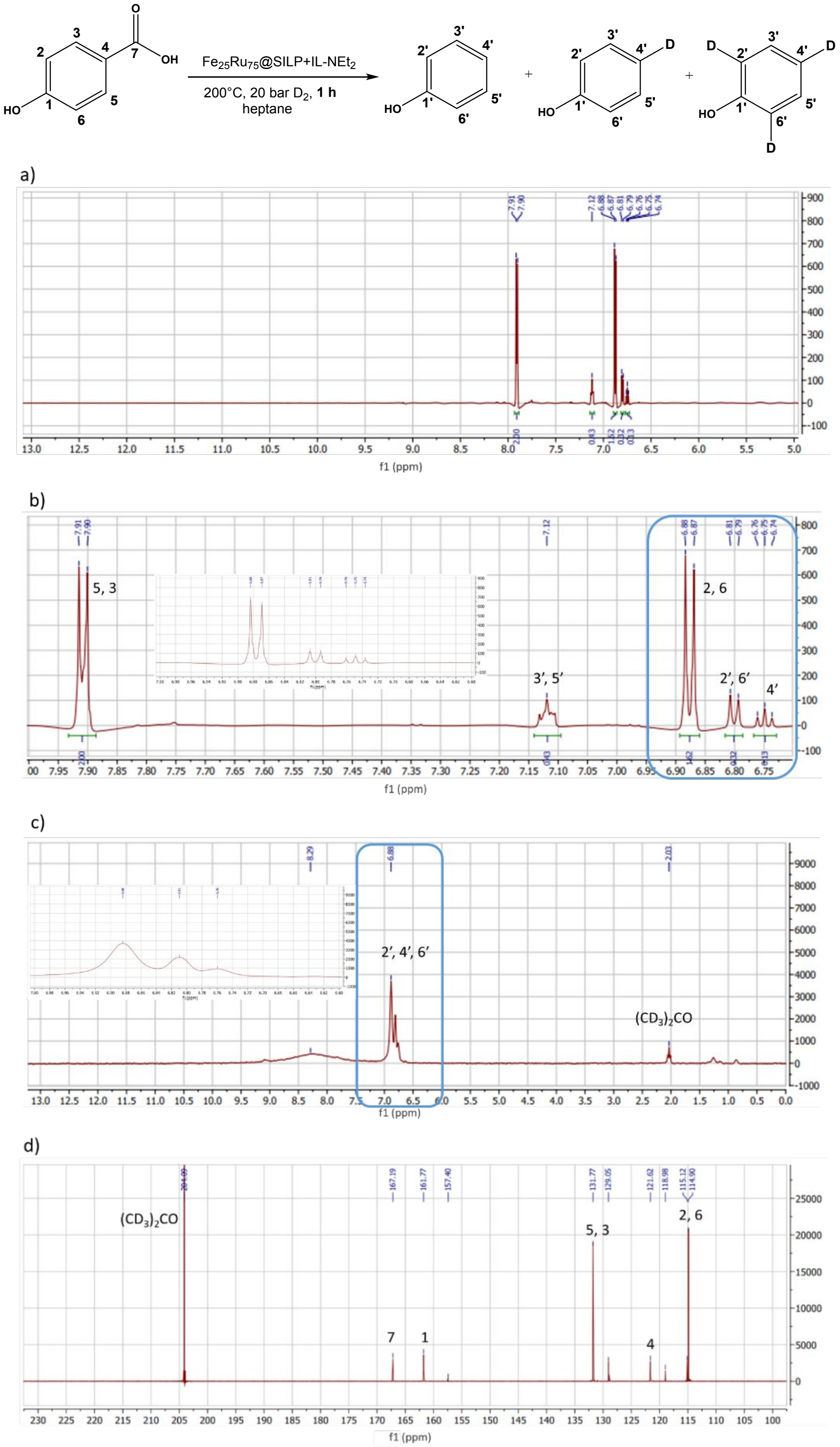

Figure S3: NMR (400 MHz, acetone-d_6) spectra of a 1 h reaction of 4-hydroxybenzoic acid under 20 bar D_2 at 200 °C. a) ^{1}H NMR spectrum, b) zoom on the aromatic area of a) with a closer look into the marked area in blue, c) ^{2}H NMR spectrum with a closer look into the marked area, d) ^{13}C NMR spectrum. Under these conditions, the ^{13}C NMR spectrum did not show the triplet associated to the deuteration in *para* position, due to the low intensity of the signal associated to the low conversion.

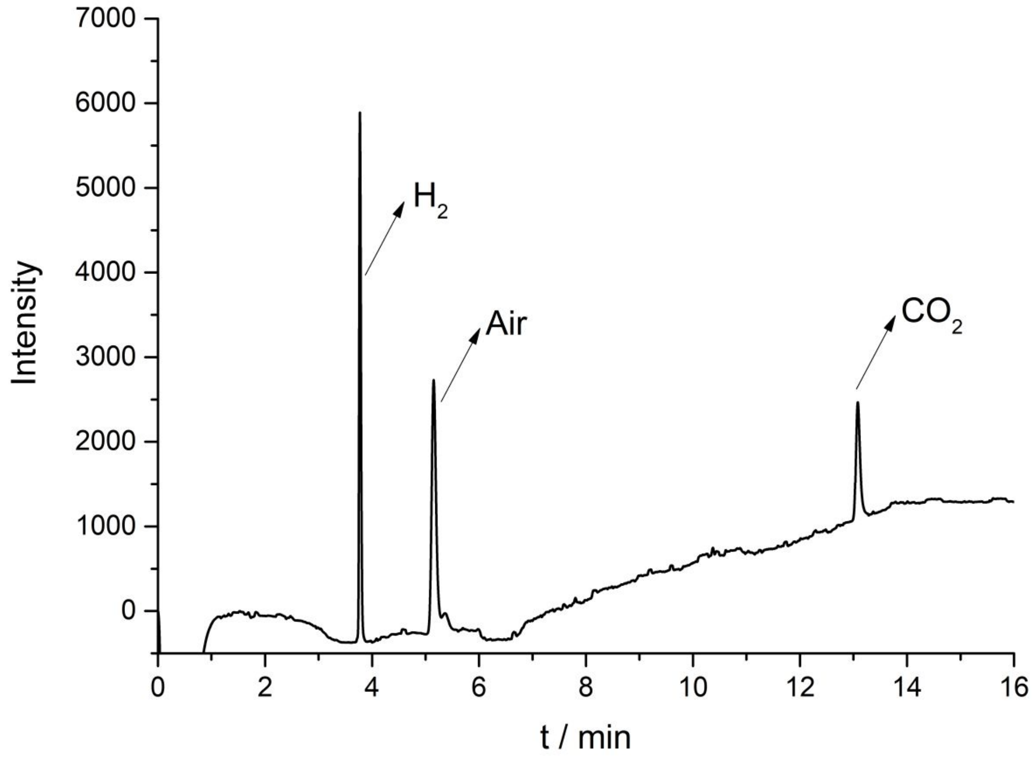

Figure S4: Gas phase GC analysis of an experiment of decarboxylation of 4-hydroxybenzoic acid under standard reaction conditions: $Fe_{25}Ru_{75}$@SILP+IL-NEt_2 (metal content: 0.0034 mmol), substrate (0.221 mmol, 65 eq. compared to metal), solvent: heptane (0.5 mL), 175 °C, H_2 (50 bar), 18 h, 500 rpm.

11.1. NMR characterization associated to isolated products

11.1.1. Phenol (**1a**)

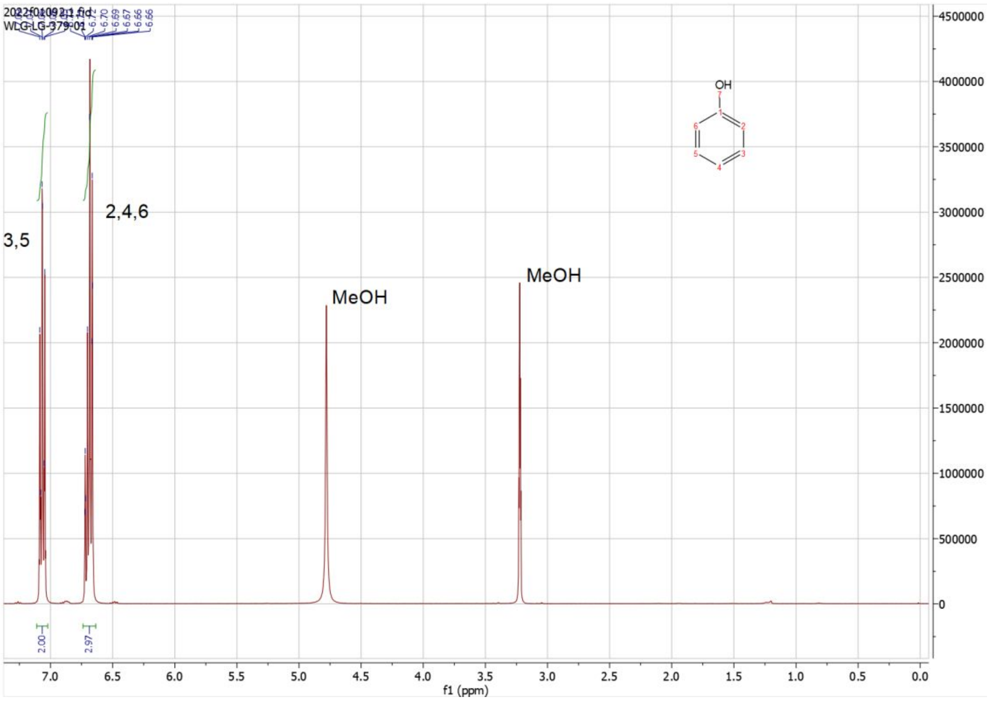

^1H NMR (400 MHz, Methanol-d_4) δ (ppm) = 7.11 – 7.02 (m, 2H), 6.74 – 6.64 (m, 3H).

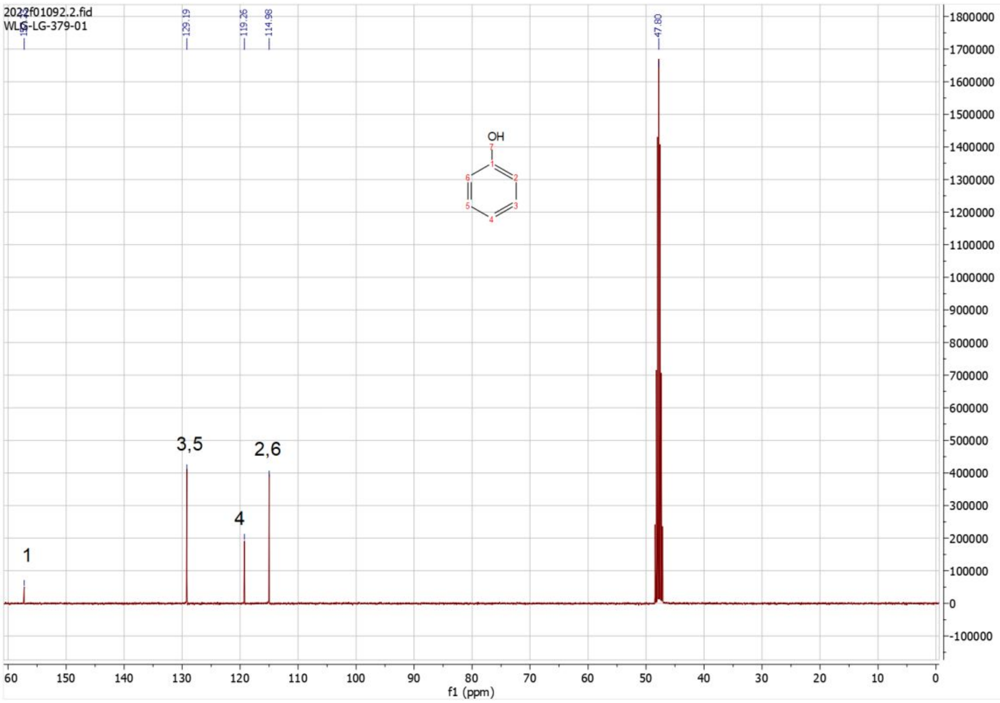

$^{13}C\{^1H\}$ NMR (100 MHz, Methanol-d_4) δ (ppm) = 157.23 (1C), 129.19 (2C), 119.26 (1C), 114.98 (2C).

11.1.2. Product isolated from exposure of phenol (**1a**) to reaction conditions under D_2

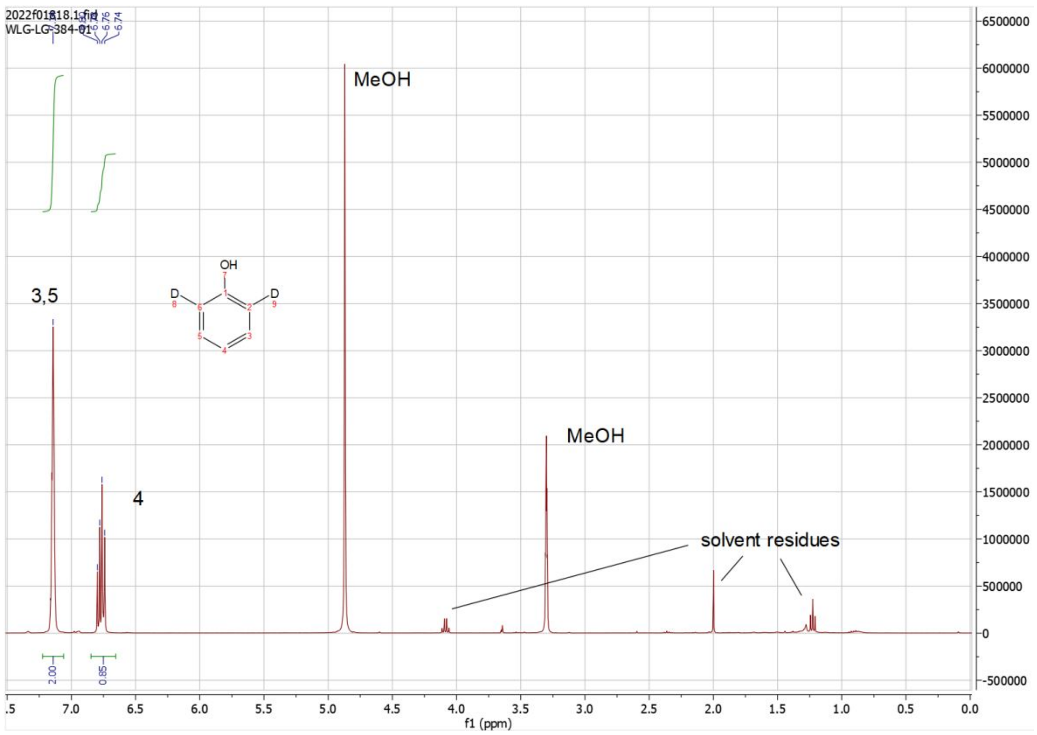

^{1}H NMR (400 MHz, Methanol-d_4) δ (ppm) = 7.14 (s, 2H), 6.85 – 6.65 (m, 1H).

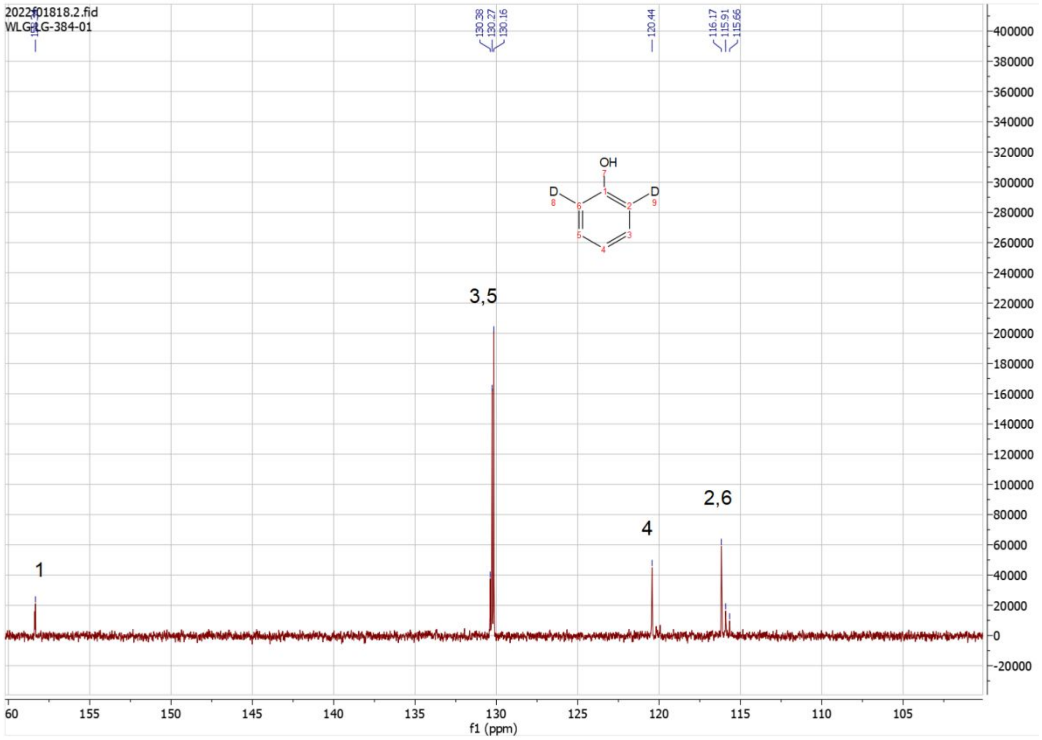

^{13}C{^{1}H} NMR (100 MHz, Methanol-d_4) δ (ppm) = 158.34 (1C), 130.27 (2C), 120.44 (1C), 115.91 (2C).

*11.1.3. Product isolated from decarboxylation of 4-hydroxybenzoic acid (**1**) under D_2*

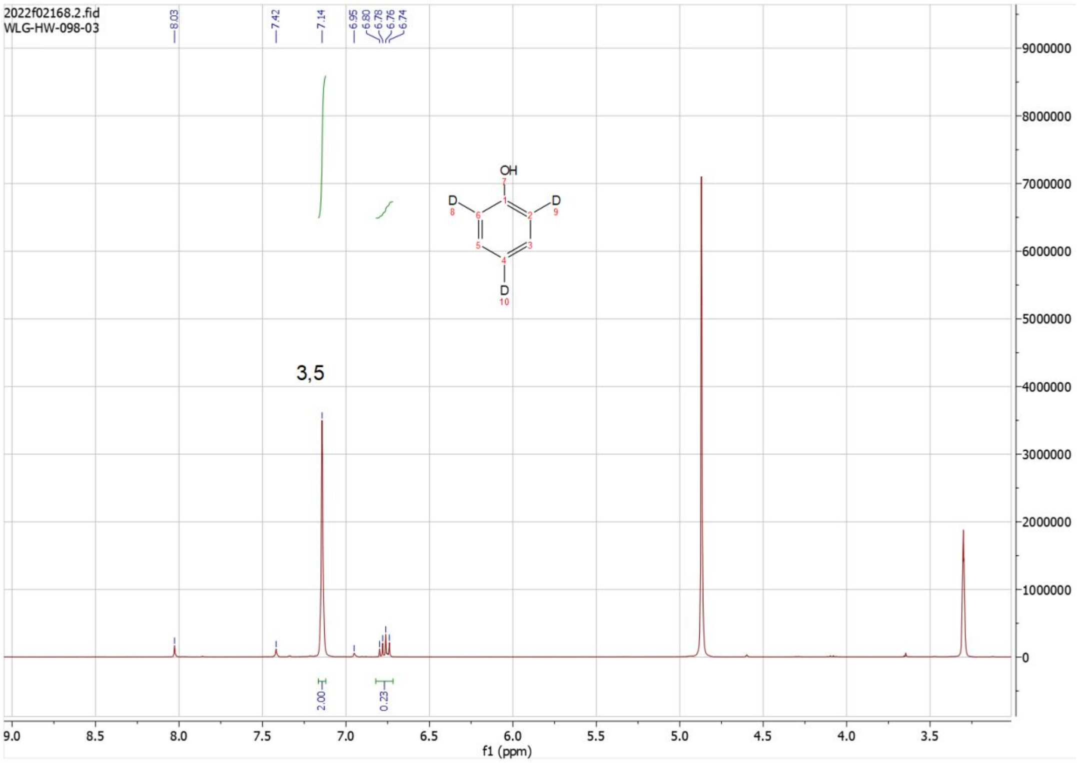

^1H NMR (400 MHz, Methanol-d_4) δ (ppm) = 7.14 (s, 2H).

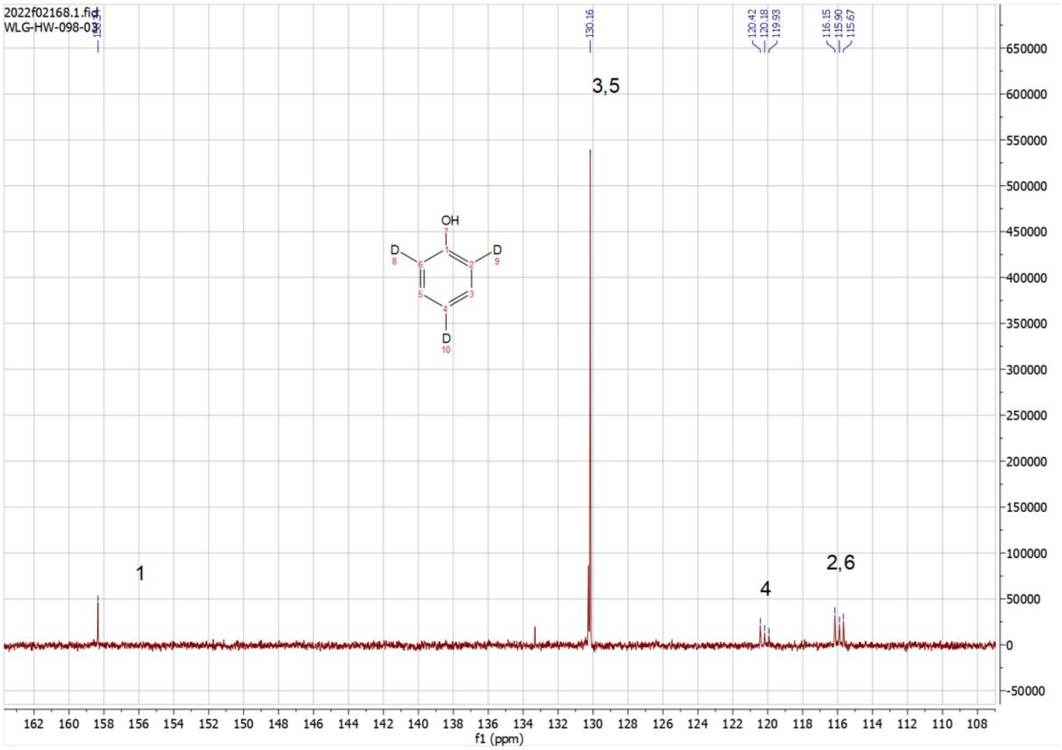

^{13}C NMR (100 MHz, Methanol-d_4) δ (ppm) = 158.34 (1C), 130.16 (2C), 120.18 (1C), 115.90 (2C).

*11.1.4. 2,6-Dimethoxyphenol (**3a**)*

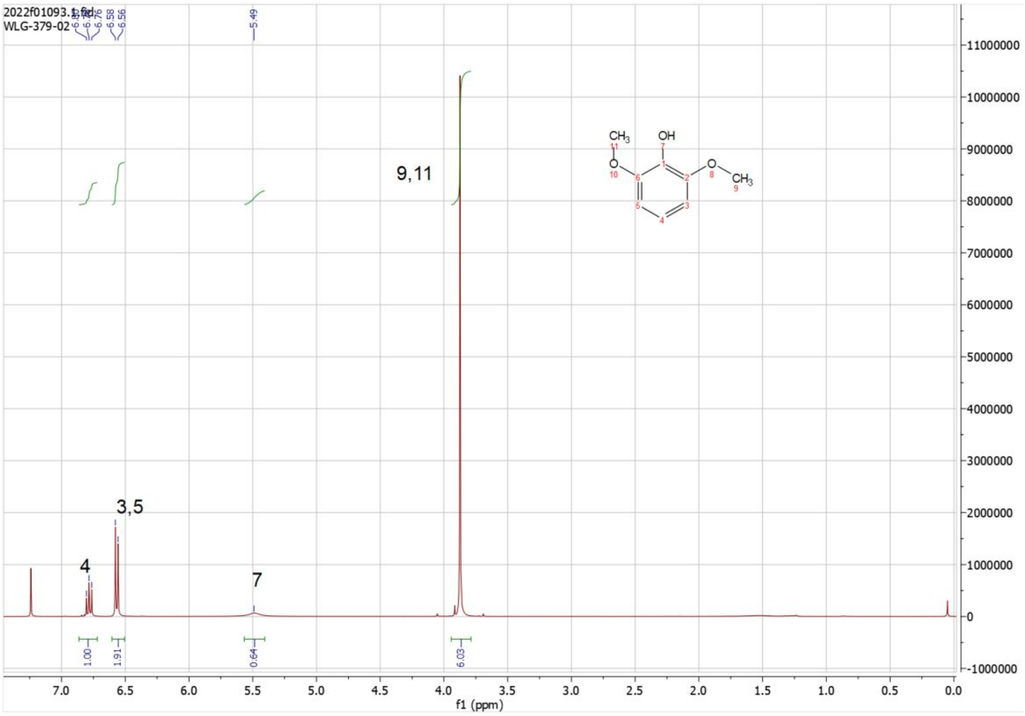

^{1}H NMR (400 MHz, Chloroform-*d*) δ (ppm) = 6.86 – 6.72 (m, 1H), 6.57 (d, J = 8.3 Hz, 2H), 5.49 (s, 1H), 3.87 (s, 6H).

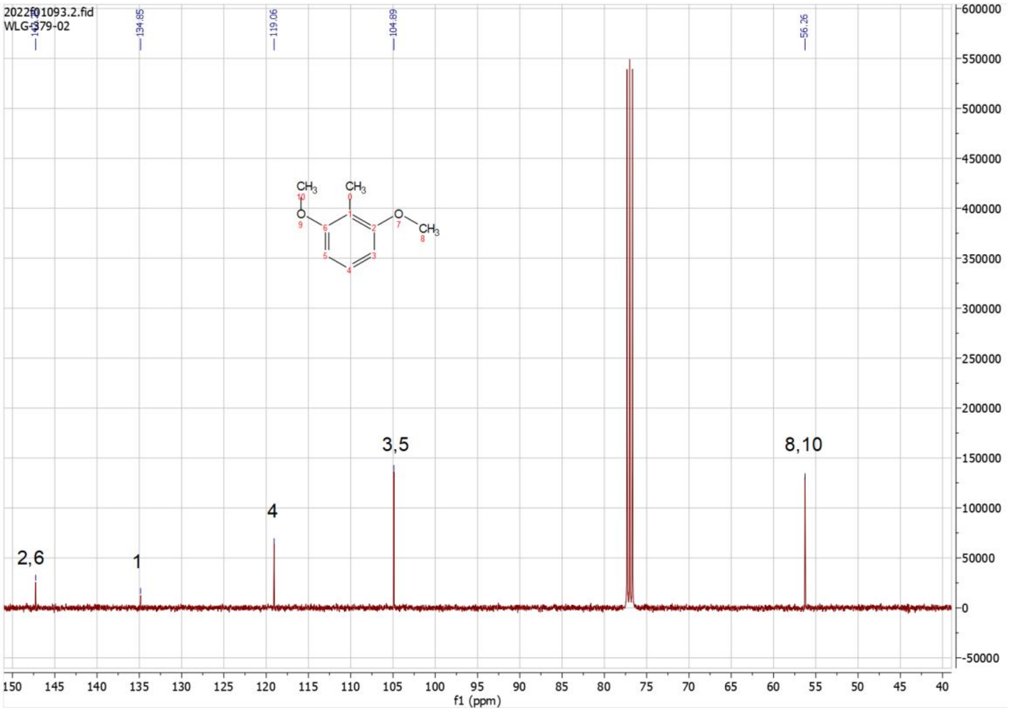

$^{13}C\{^{1}H\}$ NMR (100 MHz, Chloroform-*d*) δ (ppm) = 147.25 (2C), 134.85 (1C), 119.06 (1C), 104.89 (2C), 56.26 (2C).

11.1.5. 2-Ethylphenol (**10a**)

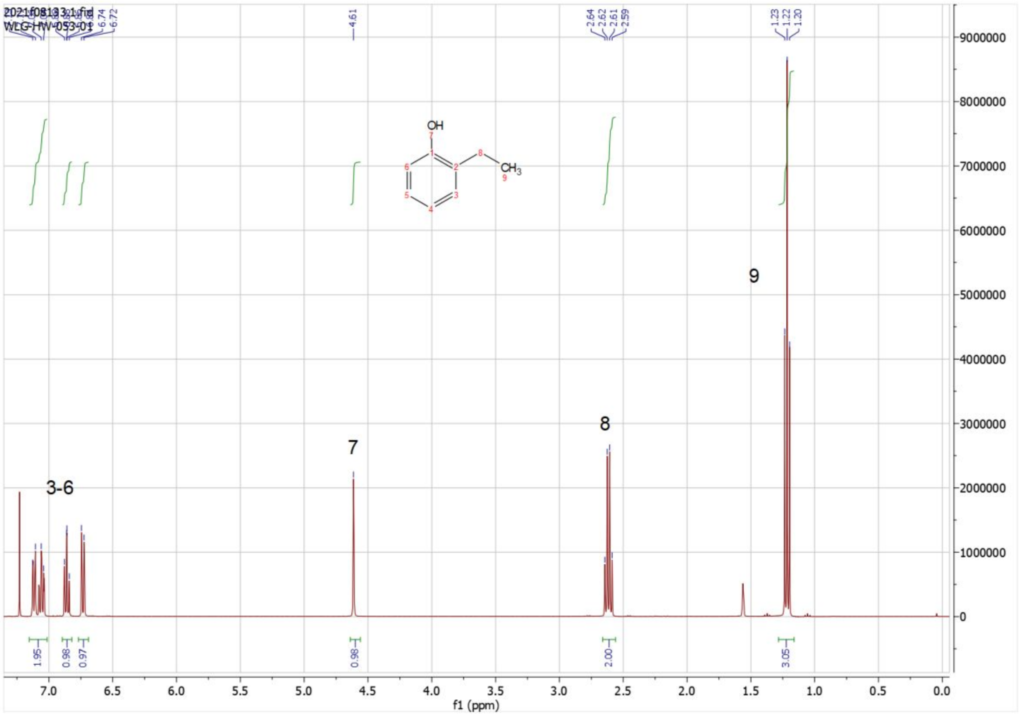

^{1}H NMR (400 MHz, Chloroform-*d*) *δ* (ppm) = 7.15 – 7.01 (m, 2H), 6.89 – 6.82 (m, 1H), 6.73 (d, *J* = 8.0 Hz, 1H), 4.61 (s, 1H), 2.62 (q, *J* = 7.6 Hz, 2H), 1.22 (t, *J* = 7.6 Hz, 3H).

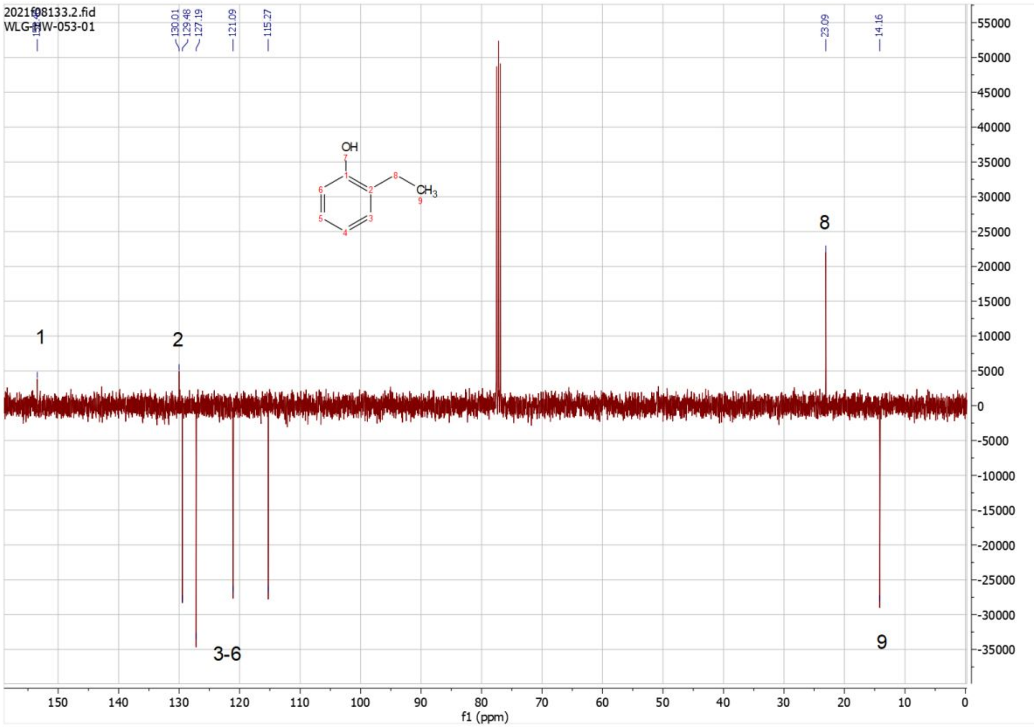

^{13}C (APT) NMR (100 MHz, Chloroform-*d*) *δ* (ppm) = 153.46 (1C), 130.01 (1C), 129.48 (1C), 127.19 (1C), 21.09 (1C), 115.27 (1C), 23.09 (1C), 14.16 (1C).

*11.1.6. 2-Aminophenol (**6a**)*

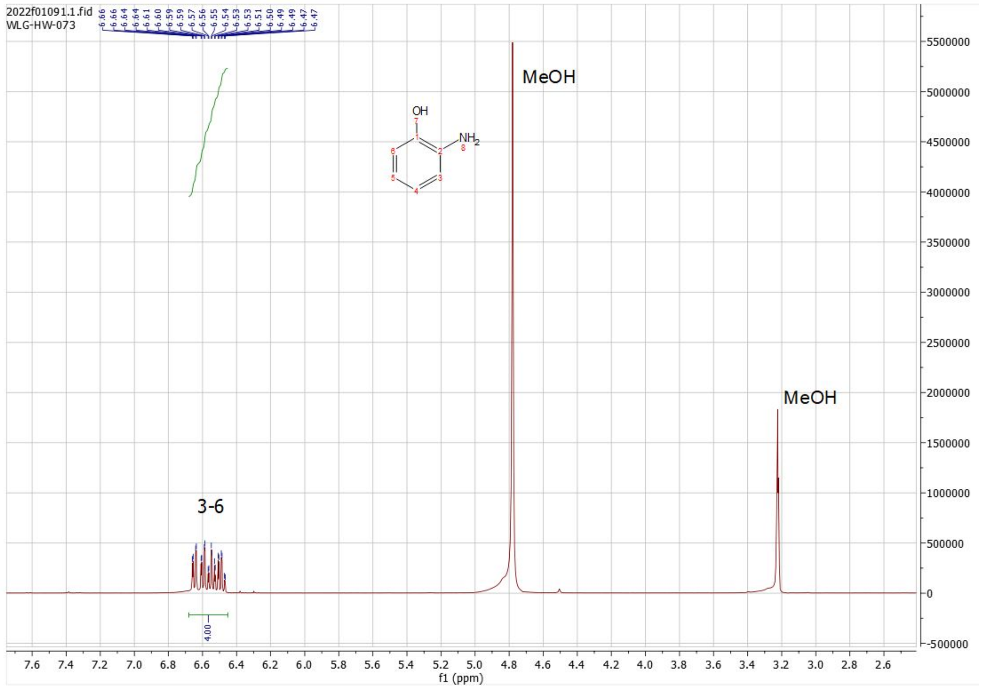

^1H NMR (400 MHz, Methanol-d_4) δ (ppm) = 6.66 – 6.47 (m, 4H).

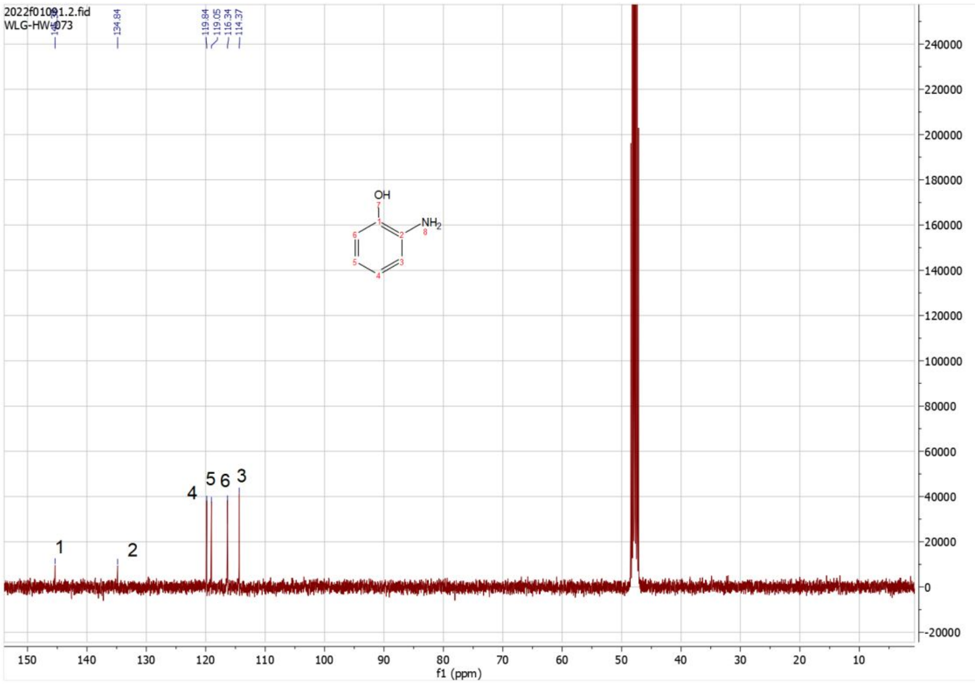

$^{13}C\{^1H\}$ NMR (100 MHz, Methanol-d_4) δ (ppm) = 145.38 (1C), 134.84 (1C), 119.84 (1C), 119.05 (1C), 116.34 (1C), 114.37 (1C).

*11.1.7. 4-Ethylphenol (**13a**)*

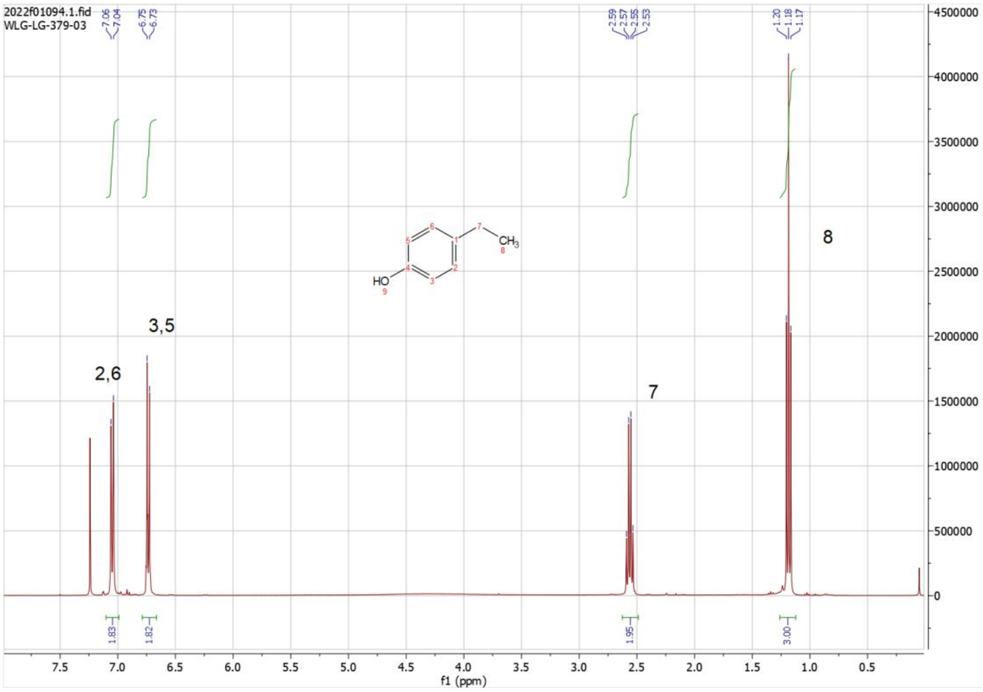

^1H NMR (400 MHz, Chloroform-*d*) *δ* (ppm) = 7.05 (d, *J* = 8.5 Hz, 2H), 6.74 (d, *J* = 8.5 Hz, 2H), 2.56 (q, *J* = 7.6 Hz, 2H), 1.18 (t, *J* = 7.6 Hz, 3H).

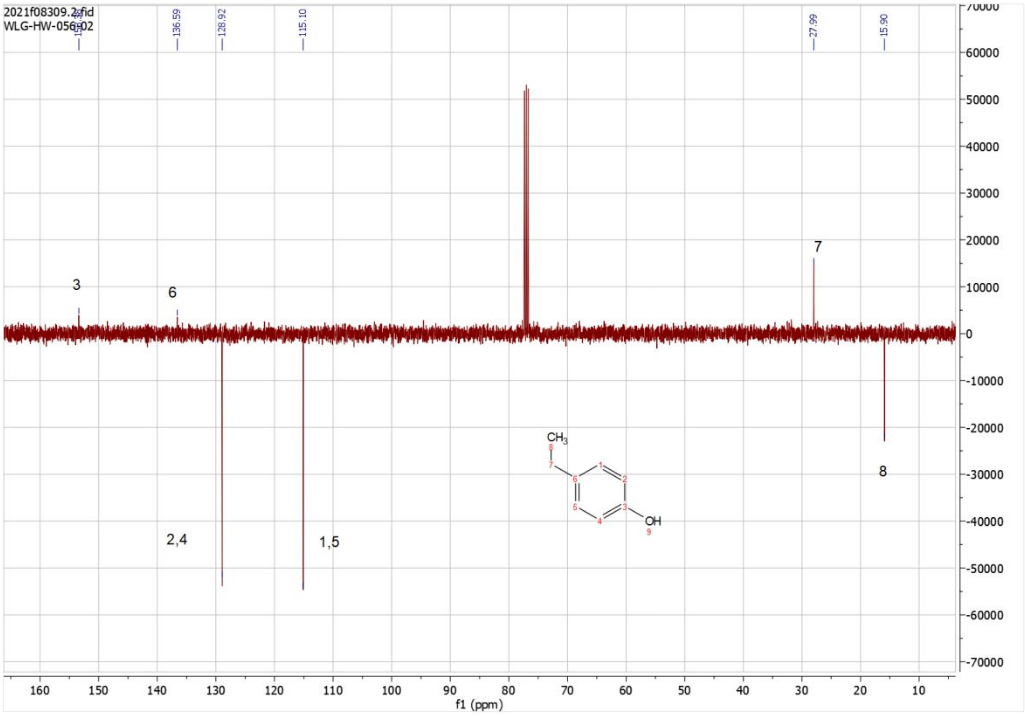

^{13}C (APT) NMR (100 MHz, Chloroform-*d*) *δ* (ppm) = 153.38 (1C), 136.59 (1C), 128.92 (2C), 115.10 (2C), 27.99 (1C), 15.90 (1C).

11.1.8. 4-Octylphenol (**14a**)

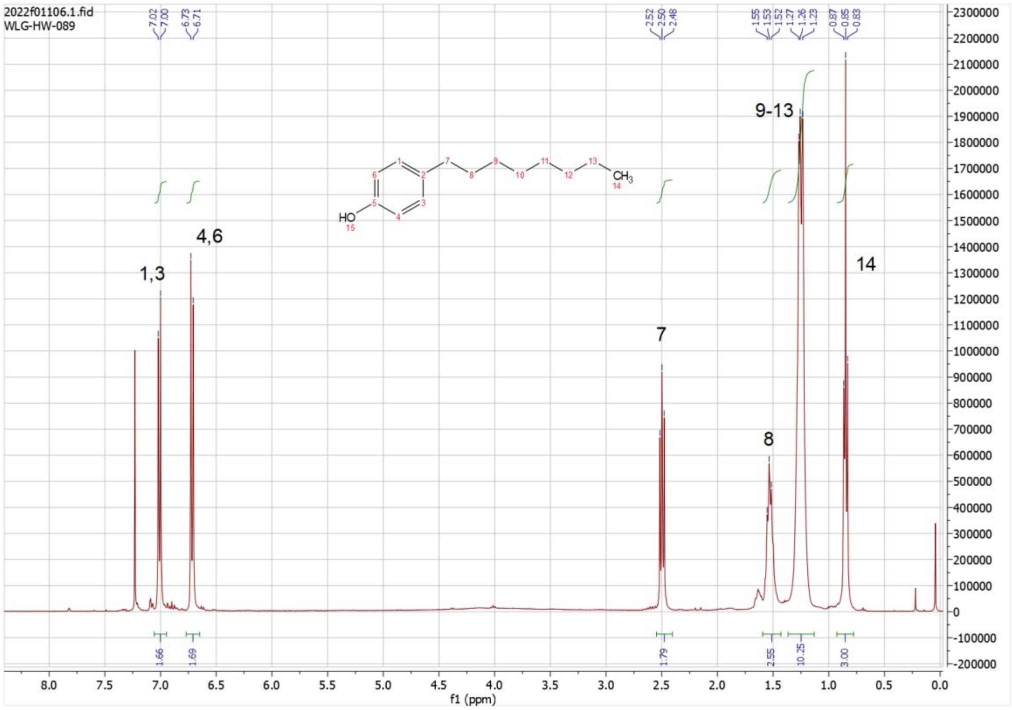

^{1}H NMR (400 MHz, Chloroform-*d*) *δ* (ppm) = 7.01 (d, *J* = 8.3 Hz, 2H), 6.72 (d, *J* = 8.4 Hz, 2H), 2.55 – 2.40 (m, 2H), 1.59 – 1.43 (m, 2H), 1.37 – 1.13 (m, 10H), 0.85 (t, *J* = 6.8 Hz, 3H).

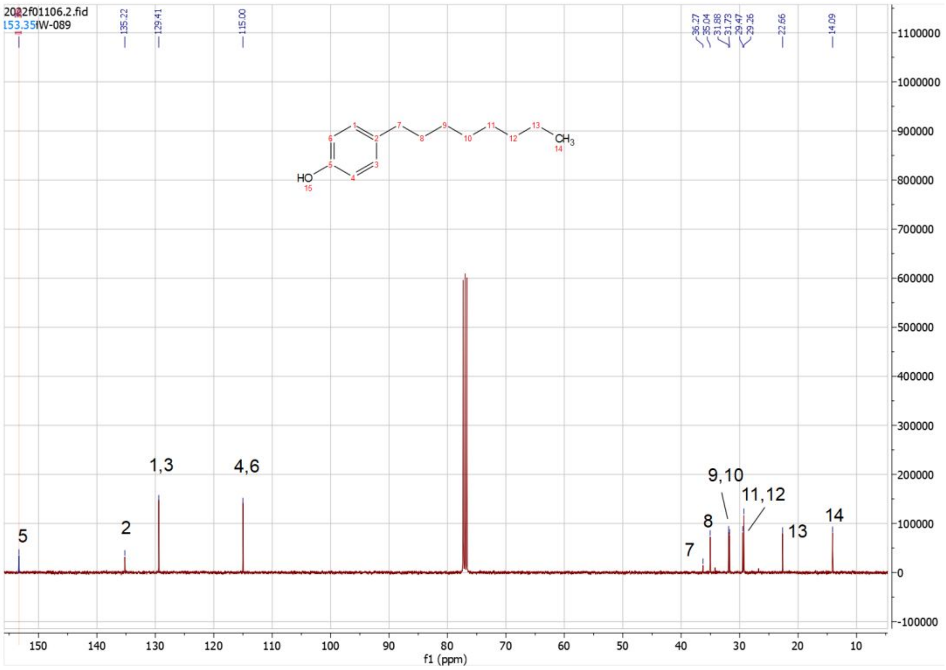

^{13}C{^{1}H} NMR (100 MHz, Chloroform-*d*) *δ* (ppm) = 153.35 (1C), 135.22 (1C), 129.41 (2C), 115.00 (2C), 26.27 (1C), 35.04 (1C), 31.88 (1C), 31.73 (1C), 29.47 (1C), 29.36 (1C), 22.66 (1C), 14.09 (1C).

11. References

(1) Boselli, L.; Ader, I.; Carraz, M.; Hemmert, C.; Cuvillier, O.; Gornitzka, H. Synthesis, Structures, and Selective Toxicity to Cancer Cells of Gold(I) Complexes Involving N-Heterocyclic Carbene Ligands. *Eur. J. Med. Chem.* **2014**, *85*, 87-94.
(2) Offner, M. L.; Bordet, A.; Moos, G.; Tricard, S.; Rengshausen, S.; Chaudret, B.; Luska, K. L.; Leitner, W. Bimetallic Nanoparticles in Supported Ionic Liquid Phases as Multifunctional Catalysts for the Selective Hydrodeoxygenation of Aromatic Substrates. *Angew. Chem., Int. Ed.* **2018**, *57*, 12721-12726.
(3) Neese, F.; Wennmohs, F.; Becker, U.; Riplinger, C. The ORCA Quantum Chemistry Program Package. *J. Chem. Phys.* ***2020**, 152, 224108*.
(4) Becke, A. D. Density-functional Exchange-Energy Approximation with Correct Asymptotic Behavior. *Phys. Rev. A* **1988**, *38*, 3098-3100.
(5) Becke, A. D. Density-functional Thermochemistry. III. The Role of Exact Exchange. *J. Chem. Phys.* **1993**, *98*, 5648-5652.
(6) Lee, C.; Yang, W.; Parr, R. G. Development of the Colle-Salvetti Correlation-Energy Formula into a Functional of the Electron Density. *Phys. Rev. B* **1988**, *37*, 785-789.
(7) Weigend, F.; Ahlrichs, R. Balanced Basis Sets of Split Valence, Triple Zeta Valence and Quadruple Zeta Valence Quality for H to Rn: Design and Assessment of Accuracy. *PCCP* **2005**, *7*, 3297-3305.
(8) Barone, V.; Cossi, M. Conductor Solvent Model. *J. Phys. Chem. A* **1998**, *102*, 1995-2001.
(9) Nahman, N. S. Dielectric Constant Measurements on *n*-Heptane and 2-Heptanone; Los Alamos, NM, **1994**.
(10) Tshepelevitsh, S.; Kütt, A.; Lõkov, M.; Kaljurand, I.; Saame, J.; Heering, A.; Plieger, P. G.; Vianello, R.; Leito, I. On the Basicity of Organic Bases in Different Media. *Eur. J. Org. Chem*. **2019**, *2019*, 6735-6748.
(11) Ho, J.; Coote, M. L. A Universal Approach for Continuum Solvent pKa Calculations: Are We There Yet? *Theor. Chem. Acc.* **2009**, *125*, 3-21.
(12) Levin, N.; Codesido, N. O.; Marcolongo, J. P.; Alborés, P.; Weyhermüller, T.; Olabe, J. A.; Slep, L. D. Remarkable Changes of the Acidity of Bound Nitroxyl (HNO) in the $[Ru(Me_3[9]aneN_3)(L^2)(NO)]^{n+}$ Family (n = 1–3). Systematic Structural and Chemical Exploration and Bioinorganic Chemistry Implications. *Inorg. Chem.* **2018**, *57*, 12270-12281.
(13) Busch, M.; Ahlberg, E.; Ahlberg, E.; Laasonen, K. How to Predict the pKa of Any Compound in Any Solvent. *ACS Omega* **2022**, *7*, *17369-71383.*
(14) Xue, L.; Su, W.; Lin, Z. Mechanism of Silver- and Copper-Catalyzed Decarboxylation Reactions of Aryl Carboxylic Acids. *Dalton Trans.* **2011**, *40*, 11926-11936.
(15) Grainger, R.; Cornella, J.; Blakemore, D. C.; Larrosa, I.; Campanera, J. M. The *ortho*-Substituent Effect on the Ag-Catalysed Decarboxylation of Benzoic Acids. *Chem. Eur. J.* ***2014**, 20, 16680-16687*.
